# Supplementary material for: Pancreatic cancer-derived extracellular vesicles enhance chemoresistance by delivering KRASG12D protein to cancer-associated fibroblasts
Source: Mol Ther. 2025 Jan 14;33(3):1134–53. doi: 10.1016/j.ymthe.2025.01.023 (PMC11897769; doi:10.1016/j.ymthe.2025.01.023)
Supplement: Document S1. Figure S1–S7 and Tables S1 [file mmc1.pdf]

YMTHE, Volume *33*

Supplemental Information

Pancreatic cancer-derived extracellular vesicles enhance chemoresistance by delivering $KRAS^{G12D}$ protein to cancer-associated fibroblasts

Xinyuan Liu, Jiaqi Yang, Sicong Huang, Yifan Hong, Yupeng Zhu, Jianing Wang, Yi Wang, Tingbo Liang, and Xueli Bai

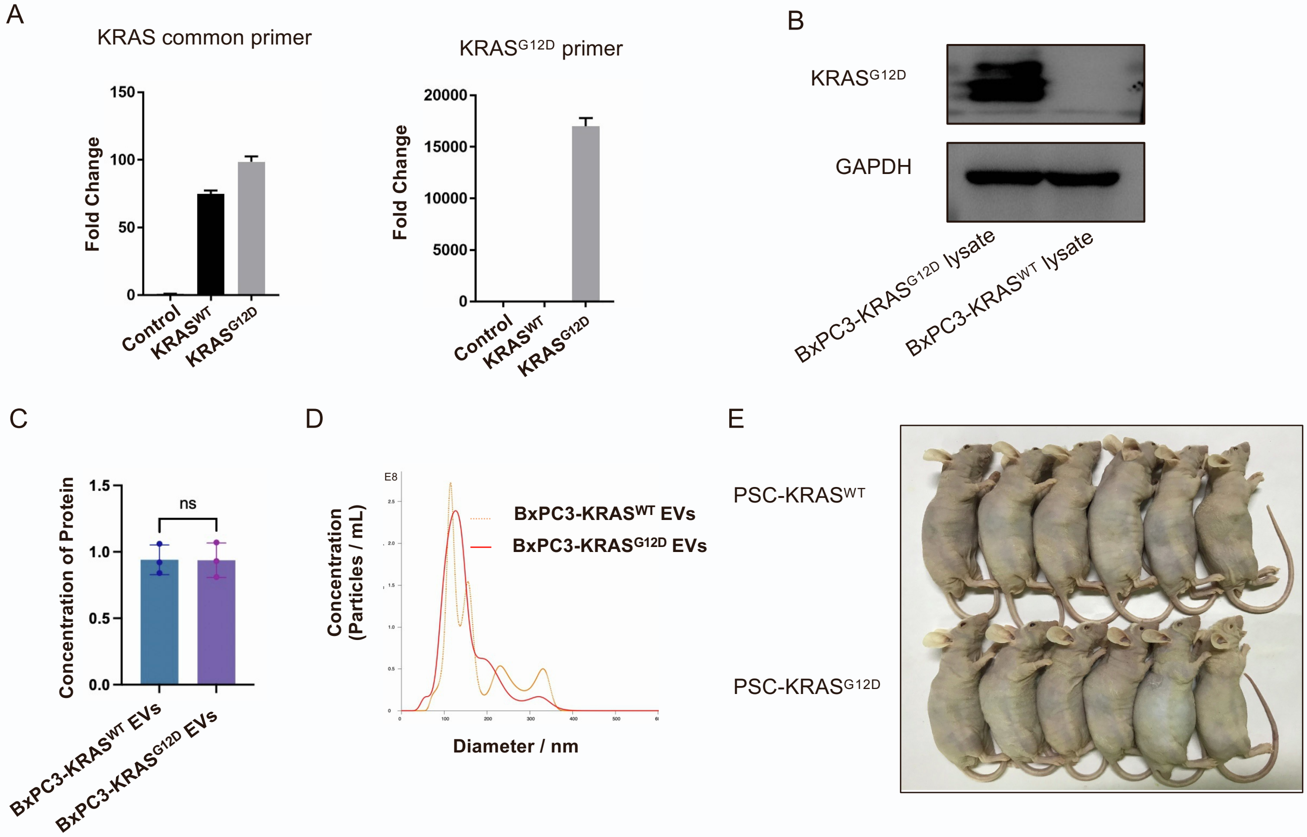

Figure S1. PSCs overexpressing $KRAS^{G12D}$ acquire biological function.

(A) Quantitative Real-time PCR of $KRAS^{WT}$ and $KRAS^{G12D}$ expression in PSC-$KRAS^{WT}$, PSC-$KRAS^{G12D}$ and control cells (n = 3). (B) Western blot analysis of $KRAS^{G12D}$ proteins in BxPC3-$KRAS^{WT}$ and BxPC3-$KRAS^{G12D}$ cells. (C) BCA analysis of protein concentration of EVs collected from 20,000,000 cells of BxPC3-$KRAS^{WT}$ and BxPC3-$KRAS^{G12D}$ cells (n = 3). (D) Nanosight analysis of EVs concentration collected from 20,000,000 cells of BxPC3-$KRAS^{WT}$ and BxPC3-$KRAS^{G12D}$ cells. (E) Representative images of nude mice bearing PSC-$KRAS^{WT}$ and PSC-$KRAS^{G12D}$ in right flank (n = 6).

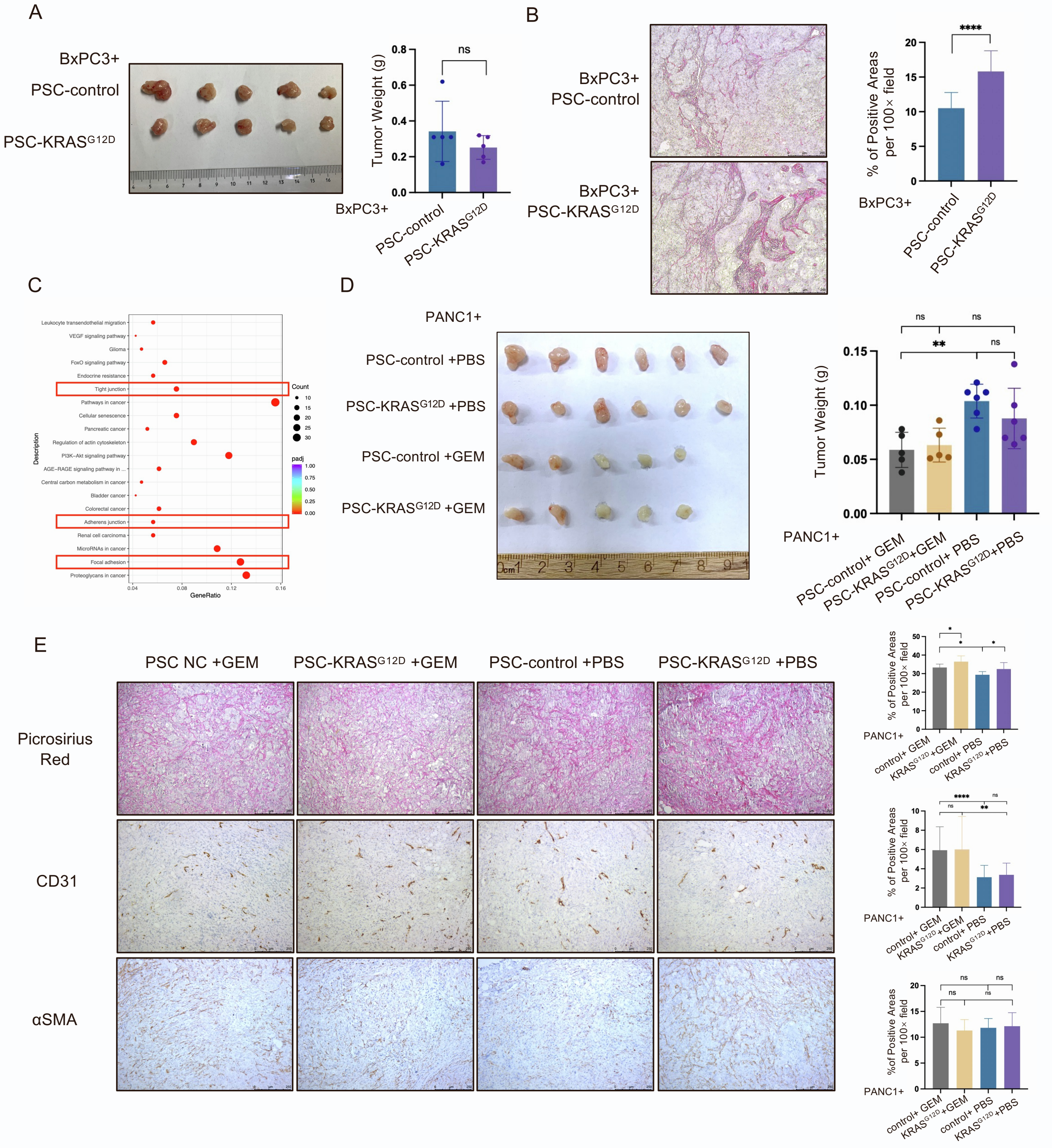
A
BxPC3+
PSC-control
PSC-KRASG12D
Tumor Weight (g)
ns
BxPC3+
PSC-control
PSC-KRASG12D
B
BxPC3+
PSC-control
BxPC3+
PSC-KRASG12D
% of Positive Areas per 100× field
****
BxPC3+
PSC-control
PSC-KRASG12D
C
Leukocyte transendothelial migration
VEGF signaling pathway
Glioma
FoxO signaling pathway
Endocrine resistance
Tight junction
Pathways in cancer
Cellular senescence
Pancreatic cancer
Regulation of actin cytoskeleton
PI3K–Akt signaling pathway
AGE–RAGE signaling pathway in ...
Central carbon metabolism in cancer
Bladder cancer
Colorectal cancer
Adherens junction
Renal cell carcinoma
MicroRNAs in cancer
Focal adhesion
Proteoglycans in cancer
Description
GeneRatio
Count
padj
D
PANC1+
PSC-control +PBS
PSC-KRASG12D +PBS
PSC-control +GEM
PSC-KRASG12D +GEM
Tumor Weight (g)
ns
ns
**
ns
PANC1+
PSC-control+ GEM
PSC-KRASG12D+GEM
PSC-control+ PBS
PSC-KRASG12D+PBS
E
PSC NC +GEM
PSC-KRASG12D +GEM
PSC-control +PBS
PSC-KRASG12D +PBS
Picrosirius Red
CD31
αSMA
% of Positive Areas per 100× field
PANC1+
control+ GEM
KRASG12D+GEM
control+ PBS
KRASG12D+PBS
%of Positive Areas per 100× field

Figure S2. PSCs overexpressing KRAS^{G12D} promote collagen deposition and increases resistance to chemotherapy.

(A) Representative images and statistical analysis of tumor in NCG mice subcutaneously bearing BxPC3 and PSC-KRAS^{G12D} or control cells (n = 5, ns: not significant, two-tailed t-test). (B) Representative picrosirius red staining images and statistical analysis of percentage of positive areas of tumor in two groups in A (n = 5, three fields of view were selected for each tumor tissue, ****P < 0.0001, two-tailed t-test) (scale bars: 100×: 50 μm). (C) KEGG pathway from RNA sequencing between PSC-KRAS^{G12D} and control cells. (D) Representative images and statistical analysis of tumor in NCG mice subcutaneously bearing PANC1 and PSC-KRAS^{G12D} or control cells administered with gemcitabine (20 mg/kg) or PBS every three days (group treated with PBS n = 6, group treated with gemcitabine n = 5). (E) Representative images and statistical results of picrosirius red staining, CD31 and αSMA of tumor in each group (group treated with PBS n = 6, group treated with gemcitabine n = 5, three fields of view were selected for each tumor tissue, *P < 0.05, **P < 0.01, ****P < 0.0001, two-tailed t-test) (scale bars: 100×: 50 μm).

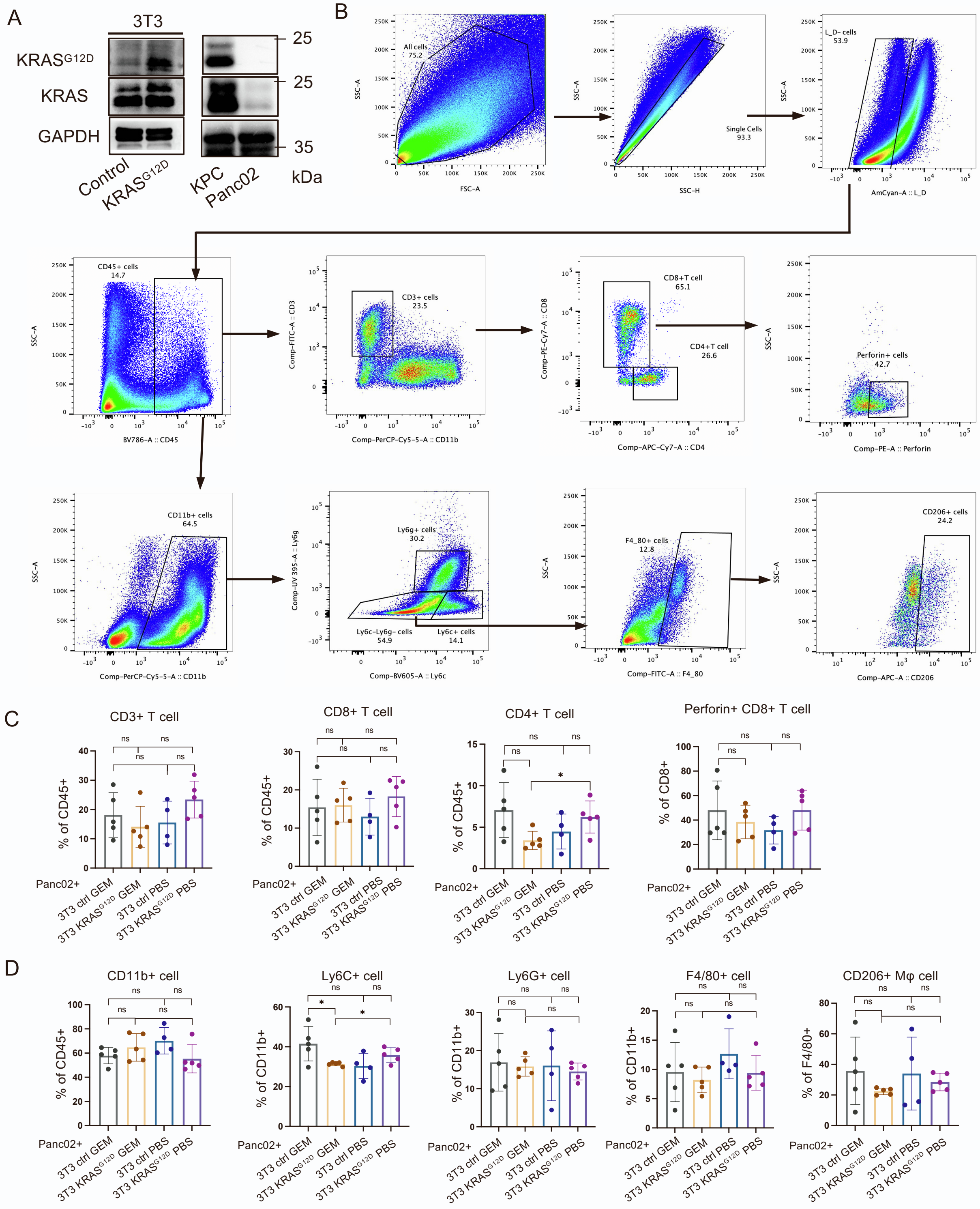

Figure S3. The overexpression of the $KRAS^{G12D}$ protein in 3T3 cells has a minimal impact on the immune cell components in tumor.

(A) Western blot analysis of $KRAS^{G12D}$ and KRAS expression in 3T3-$KRAS^{G12D}$ and control cells. (B) Representative flow cytometry dot plots of T and myeloid cells. (C) Statistics of percentage of T cells (n = 4 for Panc02 and 3T3 PBS treated group and n = 5 for other groups, $^*P < 0.05$, ns: not significant, two-tailed t-test). (D) Statistics of percentage of myeloid cells (n = 4 for Panc02 and 3T3 PBS treated group and n = 5 for other groups, ns: not significant, two-tailed t-test).

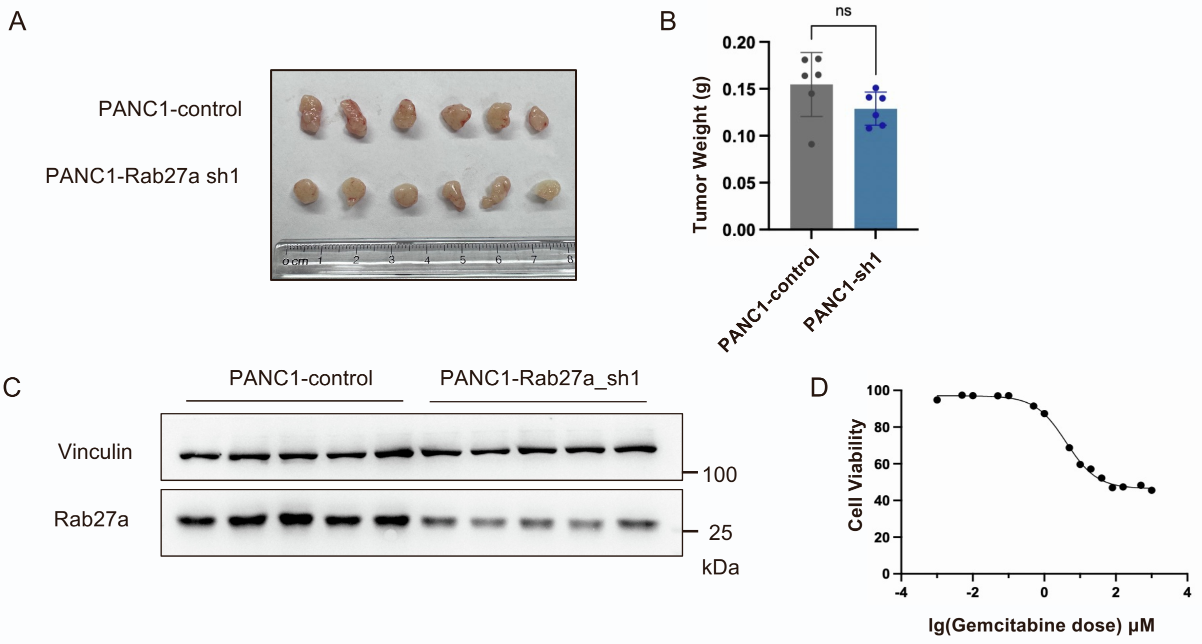

Figure S4. Blocking the secretion of EVs has no influence on tumor growth.

(A, B) Representative images and statistical results of tumor subcutaneously injected PANC1 control or PANC1-Rab27a_sh1 cell into the flank of NCG mice. (n = 6, ns: not significant, two-tailed t-test). (C) Western blot analysis Rab27a expression of tumor beared with PANC1 control or PANC1-Rab27a_sh1 cell (n = 5). (D) IC_{50} analysis of PANC1 cell treated with gemcitabine for 48 h.

A

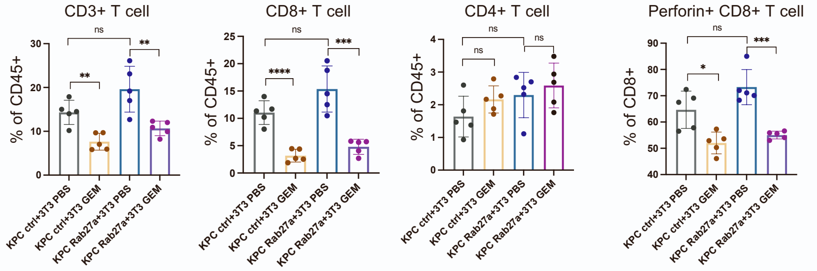

B

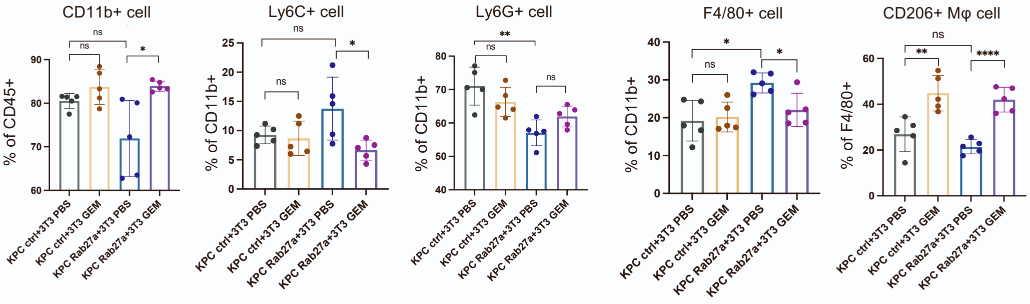

Figure S5. Reducing EVs release slightly affects specific myeloid cells within the tumor immune component.

(A) Statistics of percentage of T cells (n = 5, ***P* < 0.01, ****P* < 0.001, *****P* < 0.0001, ns: not significant, two-tailed t-test). (B) Statistics of percentage of myeloid cells (n = 5, **P* < 0.05, ***P* < 0.01, ****P* < 0.001, *****P* < 0.0001, ns: not significant, two-tailed t-test).

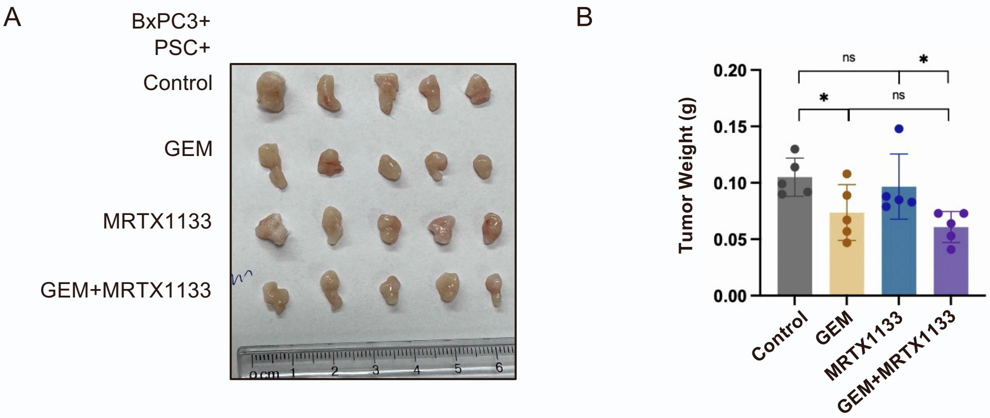

Figure S6. KRAS^{G12D} inhibitor slightly influences KRAS^{WT} tumor growth.

(A, B) Representative images and statistical results of tumor subcutaneously injected BxPC3 and PSC cells into the flank of NCG mice administered with gemcitabine (20 mg/kg), MRTX1133 (10 mg/kg) or their combination every three days (n = 5, *$P < 0.05$, ns: not significant, two-tailed t-test).

A

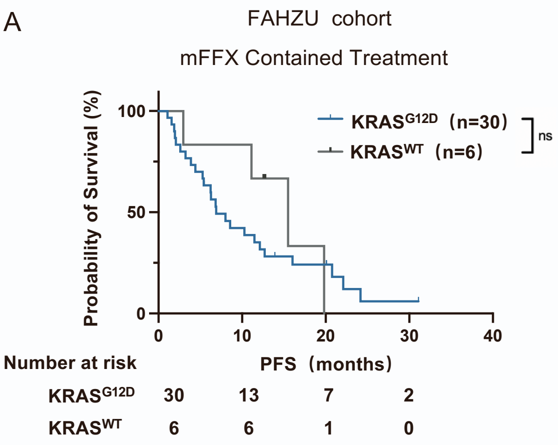

Figure S7. Patients with KRAS^{G12D} mutation have no survival influence on mFFX-containing treatment in PDAC.

(A) Survival curve of progression free survival of patients with KRAS^{G12D} mutation (n = 30) and KRAS^{WT} mutation (n = 6) who were treated with mFFX-contained regime (ns: not significant, Kaplan–Meier method and a Gehan-Breslow-Wilcoxon test).

Table S1. Basic characteristics of patients with $KRAS^{G12D}$ or $KRAS^{WT}$ mutation

	$KRAS^{G12D}$ n=59	$KRAS^{WT}$ n=15	P value
**Gender**			0.506
Male, n (%)	36(61)	9(60)	
Female, n (%)	23(39)	6(40)	
**Age, years**			0.943
>60, n (%)	37(63)	8(53)	
≤60, n (%)	22(37)	7(47)	
**BMI, kg/m2**			0.439
<18.5, n (%)	4(7)	1(7)	
18.5–23.9, n (%)	34(58)	6(40)	
>23.9, n (%)	21(36)	8(63)	
**Tumor location**			0.868
Proximal (Head, Neck or Uncinated process)	34(58)	9(60)	
Distal (Body or Tail)	25(42)	6(40)	
**Tumor size**			0.442
≥4cm, n (%)	22(37)	4(27)	
<4cm, n (%)	37(63)	11(73)	
**Lymph nodes metastasis**			0.868
Yes, n (%)	25(42)	5(33)	
No, n (%)	34(58)	10(67)	
**TNM stage**			0.235
I, n (%)	16(27)	6(40)	
II, n (%)	9(15)	3(20)	
III, n (%)	0(0)	0(0)	
IV, n (%)	34(58)	6(40)	
**Serum CA19-9, U/mL**			0.111
≥37, n (%)	46(78)	8(53)	
<37, n (%)	13(22)	7(47)	
**Serum CA12-5, U/mL**			0.656
≥60, n (%)	9(15)	1(7)	
<60, n (%)	50(85)	14(93)	
**Serum CEA, U/mL**			0.919
≥5, n (%)	19(32)	4(27)	
<5, n (%)	40(68)	11(73)	
**Recurrence**			0.217
Yes, n (%)	48(81)	10(67)	
No, n (%)	11(19)	5(33)	
