# Supplementary material for: Pancreatic cancer-derived extracellular vesicles enhance chemoresistance by delivering KRASG12D protein to cancer-associated fibroblasts
Source: Mol Ther. 2025 Jan 14;33(3):1134–53. doi: 10.1016/j.ymthe.2025.01.023 (PMC11897769; doi:10.1016/j.ymthe.2025.01.023)
Supplement: Document S2. Article plus supplemental information [file mmc2.pdf]

Molecular Therapy
Original Article

Pancreatic cancer-derived extracellular vesicles enhance chemoresistance by delivering KRAS^{G12D} protein to cancer-associated fibroblasts

Xinyuan Liu,[1,2,3,6] Jiaqi Yang,[1,2,3,4,6] Sicong Huang,[1,2,3,6] Yifan Hong,[1,2,3] Yupeng Zhu,[2,3] Jianing Wang,[1,2,3] Yi Wang,[2,3] Tingbo Liang,[1,2,3,4,5] and Xueli Bai[1,2,3,4,5]

[1]Department of Hepatobiliary and Pancreatic Surgery, The First Affiliated Hospital, Zhejiang University School of Medicine, Hangzhou, China; [2]Key Laboratory of Pancreatic Disease of Zhejiang Province, Hangzhou, China; [3]Innovation Center for the Study of Pancreatic Diseases of Zhejiang Province, Hangzhou, China; [4]Zhejiang Clinical Research Center of Hepatobiliary and Pancreatic Diseases, Hangzhou, China; [5]Cancer Center, Zhejiang University, Hangzhou, China

KRAS mutations are instrumental in the development and progression of pancreatic ductal adenocarcinoma (PDAC). Nevertheless, the efficacy of direct targeting of KRAS mutations to inhibit tumor development remains doubtful. It is therefore necessary to gain a deeper insight into the mechanism in which KRAS mutations influence the effectiveness of clinical treatments. In this study, KRAS^{G12D} protein was detected in cancer-associated fibroblasts (CAFs) from clinical samples of PDAC. *In vitro* experiments demonstrated that KRAS^{G12D} protein in CAFs was not expressed from its own mutant gene but was derived from the ingestion of tumor cell-derived extracellular vesicles (EVs). The presence of KRAS^{G12D} protein in CAFs resulted in enhanced proliferation and migration. Furthermore, KRAS^{G12D}-containing CAFs were observed to promote tumor chemoresistance to gemcitabine treatment both *in vitro* and *in vivo*. Application of a KRAS mutation-specific inhibitor, MRTX1133, has been demonstrated to reverse chemoresistance in PDAC. Moreover, clinical data suggest that patients with KRAS mutations have poorer prognosis following adjuvant chemotherapy. These findings elucidate the mechanism by which oncogenic KRAS mutations promote cancer chemoresistance and remodel tumor environment in a non-autonomous manner, suggesting a novel strategy for targeting KRAS mutations to enhance chemosensitivity in PDAC.

INTRODUCTION

Pancreatic ductal adenocarcinoma (PDAC) is an extremely malignant solid tumor that ranks fourth among all tumors in terms of mortality. It is projected that PDAC will surpass all other cancers, except lung cancer, as the leading cause of cancer-related mortality by 2040.[1] Despite the widespread implementation of promising strategies, such as immunotherapy and radiotherapy, chemotherapy remains the most effective treatment for advanced PDAC in clinical practice.[2] Gemcitabine-based combination chemotherapy represents the established standard of care as a first-line therapy.[3] However, the emergence of drug resistance significantly limits the efficacy of these treatment regimens.[4] Therefore, it is imperative to further explore the mechanisms underlying chemoresistance to improve the survival of patients with PDAC.

The presence of an abundant extracellular matrix (ECM) and a multitude of stromal cells, including cancer-associated fibroblasts (CAFs), myeloid cells, lymphocytes, and vascular endothelial cells within the highly desmoplastic extensive stroma, play a pivotal role in the advancement of malignant tumors. CAFs are pivotal elements in stromal development and interact with tumor cells through ECM components, including collagens, hyaluronic acid, fibronectin, laminin, and various chemokines and cytokines,[5] instigating a desmoplastic response in the PDAC microenvironment.[6,7] In addition, CAFs have been shown to enhance chemoresistance in many tumor types,[8,9] including PDAC.[10] Pancreatic stellate cells (PSCs), which are the precursor cells of CAFs,[11] are fibroblasts located in the periacinar, periductal, and perivascular spaces of the normal pancreas.[12] Despite the implementation of current therapeutic strategies targeting CAFs or CAF-induced ECM remodeling, including TGF-βR1 inhibition,[13] profibrotic signal inhibition,[14] and tumor stromal hyaluronan-targeted depletion,[15] their curative effects remain unsatisfactory. Therefore, it is necessary to identify more specific and efficient molecular targets of CAFs.

KRAS has received considerable attention as a frequently mutated oncogene. KRAS^{G12D}, the most prevalent KRAS mutation, is the most common mutation in pancreatic cancer, occurring in approximately 34% of the cases.[16] The RAS protein functions as a guanine

Received 4 June 2024; accepted 9 January 2025;
https://doi.org/10.1016/j.ymthe.2025.01.023.

[6]These authors contributed equally

Correspondence: Tingbo Liang, MD, PhD, FACS, Department of Hepatobiliary and Pancreatic Surgery, The First Affiliated Hospital, Zhejiang University School of Medicine, No. 79 Qingchun Road, Hangzhou 310003, China.
E-mail: liangtingbo@zju.edu.cn
Correspondence: Xueli Bai, MD, PhD, Department of Hepatobiliary and Pancreatic Surgery, The First Affiliated Hospital, Zhejiang University School of Medicine, No. 79 Qingchun Road, Hangzhou 310003, China.
E-mail: shirleybai@zju.edu.cn

 © 2025 The Authors.
Published by Elsevier Inc. on behalf of The American Society of Gene and Cell Therapy.
This is an open access article under the CC BY license (http://creativecommons.org/licenses/by/4.0/).

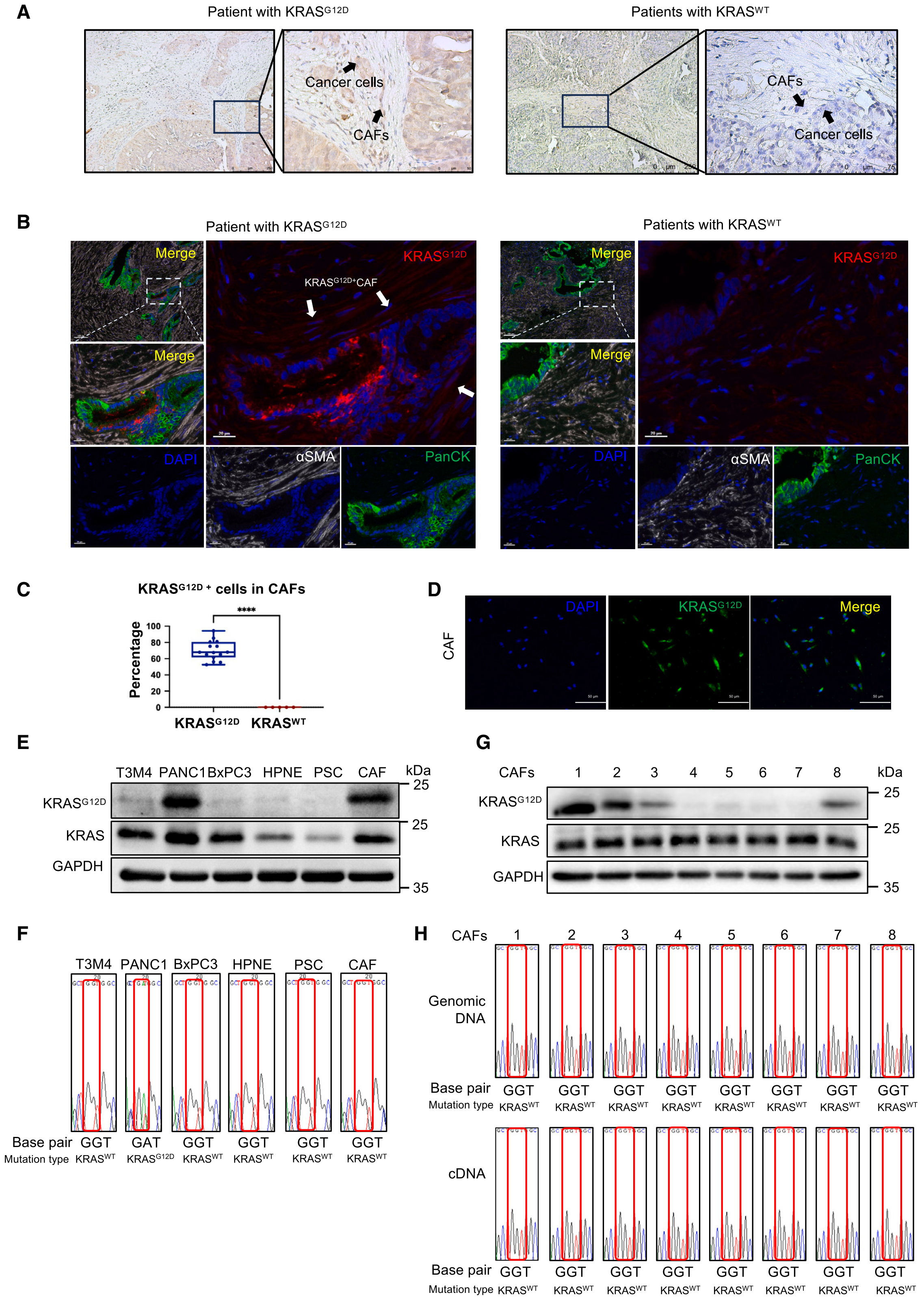

(legend on next page)

nucleotide-binding protein, acting as a binary switch between guanosine diphosphate (GDP) and triphosphate (GTP).[17] In the absence of mutations, KRAS is activated by upstream EGFR signaling, whereby it binds to GTP and activates multiple downstream signaling pathways, including the MAPK and PI3K-AKT pathways. Following GTP hydrolysis to GDP, KRAS binds to GDP and enters a resting state until reactivation occurs through further stimulation. However, when the 12th amino acid is mutated from glycine to aspartate, wild-type KRAS undergoes a transformation into $KRAS^{G12D}$, which exhibits a reduced GTP hydrolysis function. This results in the continuous activation of KRAS and the promotion of tumor progression.[18] KRAS mutations contribute to tumor progression and matrix remodeling in PDAC through interactions between tumor cells and their microenvironment.[19,20] Oncogenes within tumor cells induce aberrant signaling, both autonomously within the cells themselves and nonautonomously in adjacent stromal cells. It has been demonstrated that oncogenic KRAS is released by ferroptotic tumor cells, which drives tumor-associated macrophage polarization.[21] Moreover, in PDAC, a well-established reciprocal signaling axis has been identified between tumor cells and heterotypic CAFs.[19] It remains unclear whether oncogenic KRAS plays a role in CAF-mediated tumor progression and chemoresistance in PDAC.

In this study, mutant $KRAS^{G12D}$ protein was detected in CAFs derived from human PDAC samples. Extracellular vesicles (EVs), which are secreted by various cell types, carry nucleic acids and proteins that exhibit significant heterogeneity and play a crucial role in intercellular communication. It was demonstrated that EVs derived from tumor cells and containing oncogenic $KRAS^{G12D}$ protein can be internalized by CAFs. Subsequently, $KRAS^{G12D}$ stimulates CAFs to exhibit increased proliferation and migration by activating downstream pathways of KRAS. Moreover, $KRAS^{G12D}$-containing CAFs were found to contribute to the chemoresistance of PDAC tumor cells in both *in vitro* and *in vivo* models. Finally, the selective $KRAS^{G12D}$ mutation inhibitor MRTX1133 has been demonstrated to reverse chemoresistance induced by CAFs containing mutant KRAS protein.

RESULTS

CAFs harbor non-endogenous $KRAS^{G12D}$ mutation proteins

It was previously assumed that oncogene mutations were exclusive to neoplastic cells and were absent in stromal cells.[22] However, PDAC is distinguished by a considerable population of CAFs, which demonstrates a robust proliferative capacity comparable with that of tumor cells.[23] To investigate the presence of oncogene mutations, particularly the highly prevalent $KRAS^{G12D}$ mutation, in pancreatic CAFs, DNA sequencing was performed on a series of clinical samples to ascertain whether the patients in question had been harboring $KRAS^{G12D}$ mutations. Immunohistochemistry and multiple immunohistochemistry (mIHC) analyses were conducted to determine the presence of $KRAS^{G12D}$ in the PDAC samples. $KRAS^{G12D}$ antigen displayed intense staining in both neoplastic cells and adjacent CAFs within the sample (Figures 1A and 1B). Moreover, the proportion of $KRAS^{G12D}$-positive CAFs was markedly elevated, accounting for 70% of the total population. In contrast, samples with wild-type KRAS exhibited no positive staining for $KRAS^{G12D}$ protein in either neoplastic cells or CAFs (Figures 1A–1C). Subsequently, CAFs were isolated from fresh tumor specimens obtained from patients with PDAC. Immunofluorescence staining was conducted to further validate the presence of $KRAS^{G12D}$ protein in CAFs (Figure 1D). A pancreatic cancer cell line displayed differential expression of KRAS and $KRAS^{G12D}$. Both PANC1 cells and CAFs expressed $KRAS^{G12D}$ (Figure 1E). Furthermore, genomic sequencing revealed the absence of $KRAS^{G12D}$ mutations at the DNA level in the isolated CAFs (Figure 1F). Subsequently, eight additional CAF lines were isolated from tumors across different patients. Of the eight primary CAF lines examined, four expressed the $KRAS^{G12D}$ mutant protein (Figure 1G). However, no mutations were identified in KRAS upon sequencing of cDNA derived from the reverse transcription of mRNA isolated from these CAFs (Figure 1H). This excluded the possibility of an exogenous mutant nucleic acid transition in the studied population. These findings demonstrate that CAFs in PDAC present with the $KRAS^{G12D}$ mutant at the protein level; however, they do not possess genomic KRAS mutations.

EVs derived from tumor cells carry the $KRAS^{G12D}$ protein and can be internalized by CAFs

We investigated the differential expression of $KRAS^{G12D}$ at both the genomic and protein levels in CAFs. We postulated that the KRAS mutant protein in CAFs is transferred either as a soluble entity or encapsulated within EVs derived from tumor cells. The role of EVs in intercellular communication and their transport between tumor cells and CAFs has been confirmed in pancreatic cancer.[21] In this context, we hypothesized that tumors release EVs containing $KRAS^{G12D}$ into CAFs, which directly incorporate the mutant KRAS protein. To test this hypothesis, we isolated EVs from PANC1 and BxPC3 cell lines by sequential centrifugation with increasing gravitational force to eliminate cellular debris and apoptotic bodies, followed by ultracentrifugation at 100K (100,000 $\times$ *g*). The purity of these EVs was verified using electron microscopy and nanoparticle tracking analysis (NTA), revealing a peak diameter of approximately 100 nm (Figures 2A and 2B). Western blot analysis demonstrated

Figure 1. $KRAS^{G12D}$ protein can be detected in CAFs from clinical PDAC samples

(A) Representative IHC images of $KRAS^{G12D}$ expression measured in $KRAS^{G12D}$ and $KRAS^{WT}$ patients' pancreatic tumor tissues. Scales bars 250 μm (100×) and 50 μm (400×). (B) Representative images of multiple IHC (mIHC) staining with DAPI, αSMA, PanCK, and $KRAS^{G12D}$ in patient's pancreatic tumor tissues with $KRAS^{G12D}$ and $KRAS^{WT}$. Scales bars, 100 μm (200×) and 20 μm (400×). White arrow: $KRAS^{G12D}$-positive CAF. (C) Statistical results of percentage of $KRAS^{G12D}$-positive cells in αSMA+ CAFs ($KRAS^{G12D}$: $n = 15$; $KRAS^{WT}$: $n = 5$, ****$p < 0.0001$, two-tailed t test. White arrows: $KRAS^{G12D}$-positive CAFs). (D) Representative immunofluorescence images of $KRAS^{G12D}$ protein levels in CAFs. Scale bars, 50 μm (400×). (E) Western blot analysis of $KRAS^{G12D}$ and KRAS proteins in the pancreatic cancer cell lines. (F) Genomic DNA sequencing of $KRAS^{G12D}$ expression in pancreatic cancer cell lines. (G) Western blot analysis of $KRAS^{G12D}$ and KRAS proteins in primary CAFs isolated from tumor tissues of eight patients. (H) Genomic DNA and cDNA sequencing of $KRAS^{G12D}$ gene expression in primary CAFs isolated from pancreatic tumor tissues of patients.

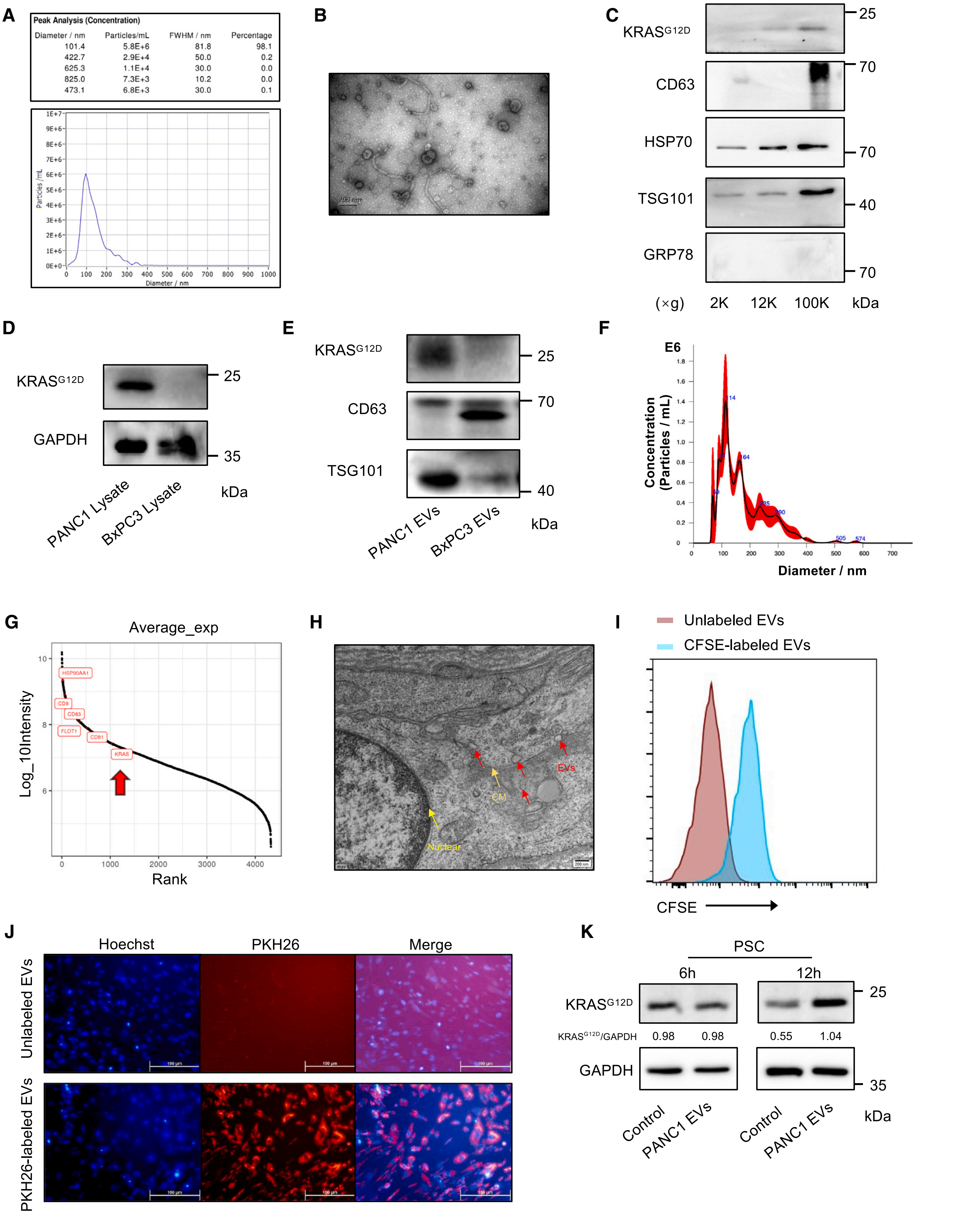

(legend on next page)

that, among sediments obtained after 2K, 12K, and 100K centrifugation (representing apoptotic bodies, cellular debris, and EVs), $KRAS^{G12D}$ protein along with typical positive markers for EV identification, including CD63, HSP70, and TSG101, were detected exclusively following the 100K centrifugation step according to the guidelines set forth by the International Society for Extracellular Vesicles. GRP78 served as a negative control for the EVs (Figure 2C). Mutant $KRAS^{G12D}$ protein was detected in EVs derived from PANC1 cells harboring the $KRAS^{G12D}$ mutation, but not in those derived from BxPC3 cells with a wild-type KRAS background (Figures 1E, 2D, and 2E). Furthermore, we isolated EVs from paired tumor and normal pancreatic tissue samples obtained from nine patients. Their purities were verified by NTA (Figure 2F). Mass spectrometric analysis of clinical tissue-derived EVs revealed detectable levels of KRAS protein within these EVs (Figure 2G). Subsequent electron microscopy further demonstrated that tumor cells could secrete EVs into the ECM. (Figure 2H). These findings suggest that KRAS may be released into the ECM through EVs originating from tumor cells.

PSCs are recognized as precursor cells of CAFs[11] and have been extensively used in CAF research.[24,25] However, our attempts to continuously culture CAFs isolated from patients in subsequent experiments have encountered significant challenges. In addition, given the potential genetic heterogeneity among CAFs derived from different patients, a comparative analysis was deemed inappropriate. Consequently, PSCs were selected as the preferred cell line for further investigation.

To elucidate the ability of CAFs to internalize oncogenic mutant KRAS proteins via EVs, we incubated carboxyfluorescein succinimidyl ester (CFSE)- and PKH26-labeled EVs with PSCs for 12 h. Flow cytometry and immunofluorescence assays confirmed the uptake of fluorescently labeled EVs by PSCs (Figures 2I and 2J). Furthermore, western blot analysis revealed significantly upregulated levels of $KRAS^{G12D}$ protein in PSCs treated with EVs derived from PANC1 cells after an incubation period of 12 h (Figure 2K). Collectively, these results provide compelling evidence that CAFs can sequester tumor cell-derived EVs containing $KRAS^{G12D}$.

The $KRAS^{G12D}$ protein augments the proliferation and migration of CAFs while activating KRAS-associated signaling pathways

Subsequently, we investigated the potential impact of $KRAS^{G12D}$ on the biological behavior of CAFs. To simulate exogenous KRAS protein in PSCs, we generated PSC-$KRAS^{WT}$ and PSC-$KRAS^{G12D}$ cell lines by stable transfection with $KRAS^{WT}$ and $KRAS^{G12D}$ plasmids, respectively (Figures 3A and S1A). In addition, BxPC3-$KRAS^{G12D}$ and BxPC3-$KRAS^{WT}$ cell lines were established (Figure 3B). Equal concentrations of proteins and numbers of EVs containing the relevant proteins were ensured for PSCs stimulation (Figures S1B–S1D). Considering that KRAS is a key signaling molecule in the MAPK pathway,[26] its elevated expression activates downstream signaling proteins.[18] Western blot analysis revealed the upregulation of downstream effectors of the MAPK pathway, including phosphorylated C-RAF (p-C-RAF) and phosphorylated ERK (p-ERK). In addition, PI3K/AKT pathway-related proteins such as phosphorylated AKT (p-AKT) and PI3K-110α were also found to be upregulated in PSCs overexpressing $KRAS^{G12D}$ or PSCs treated with EVs derived from PANC1 cells carrying the $KRAS^{G12D}$ mutation, indicating activation of downstream pathways mediated by KRAS (Figure 3C).

We hypothesized that the presence of $KRAS^{G12D}$ in PSCs confers a tumor-like phenotype, characterized by uncontrolled proliferation, enhanced migratory capacity, and increased tumorigenic potential. Our findings revealed a significant increase in the viability of PSC-$KRAS^{G12D}$ compared with that of both PSC-$KRAS^{WT}$ and control cell lines (Figure 3D). These observations were further confirmed when PSCs were treated with EVs derived from the BxPC3-$KRAS^{G12D}$ and BxPC3-$KRAS^{WT}$ cell lines (Figure 3E). Moreover, $KRAS^{G12D}$ significantly augmented the migratory ability of PSCs, as evidenced by the increased number of migrating cells after 48 h (Figures 3F–3H). In addition, treatment with EVs containing $KRAS^{G12D}$ from PANC1 cells resulted in significantly greater motility of PSCs compared with non-EV-treated controls at both 24 and 48 h (Figures 3I–3K). However, subcutaneous inoculation of both PSC-$KRAS^{G12D}$ and PSC-$KRAS^{WT}$ cells in nude mice did not result in visible tumor formation within 30 days (Figure S1E). These results suggest that, whereas $KRAS^{G12D}$ enhances the proliferative and migratory capabilities of PSCs, it does not confer tumorigenic properties similar to those observed in cancer cells.

CAFs harboring $KRAS^{G12D}$ protein facilitate collagen deposition and confer chemoresistance in PDAC

To gain further insight into the influence of PSCs on tumor growth following the incorporation of $KRAS^{G12D}$ protein, an *in vivo* experiment was conducted. This involved co-injection of KRAS wild-type BxPC3 cells with either PSC-control or PSC-$KRAS^{G12D}$ into the right flank of NCG mice. No significant differences in tumor proliferation were observed between the two groups (Figure S2A). Subsequent histological analyses revealed that tumors containing PSC-$KRAS^{G12D}$ exhibited a markedly thicker ECM (Figure S2B), whereas no apparent differences were noted in microvessel-related markers, such as CD31 (not shown). In addition, Col1A1 (alpha-1 type I collagen), a member of group I collagens, showed elevated expression levels in the presence

Figure 2. Mutant KRAS protein could be transferred to PSCs by EVs from pancreatic cancer cells

(A and B) Nanosight analysis and representative electron microscopy images of EVs extracted from the PANC1 cell line. (C) Western blot analysis of $KRAS^{G12D}$ protein and identified markers in EVs and pellets after different speed centrifugation in 2K, 12K, and 100K (×*g*). (D) Western blot analysis of $KRAS^{G12D}$ protein in the PANC1 and BxPC3 cell lines. (E) Western blot analysis of identified marker proteins in EVs derived from PANC1 and BxPC3 cells. (F) Nanosight analysis of EVs extracted from the pancreatic tumor tissues of patients. (G) LS-MS/MS analysis of EVs extracted from the pancreatic tumor tissues of patients (n = 9). (H) Representative electron microscopy images of EVs extracted from pancreatic tumor tissues of patients. (I) Flow cytometric analysis of PSCs treated with CFSE-labeled EVs for 12 h. (J) Representative immunofluorescence images of PSCs treated with PKH26-dyed EVs for 12 h. (K) Western blot analysis of $KRAS^{G12D}$ protein in PSCs treated with EVs derived from PANC1 for 6 and 12 h, respectively.

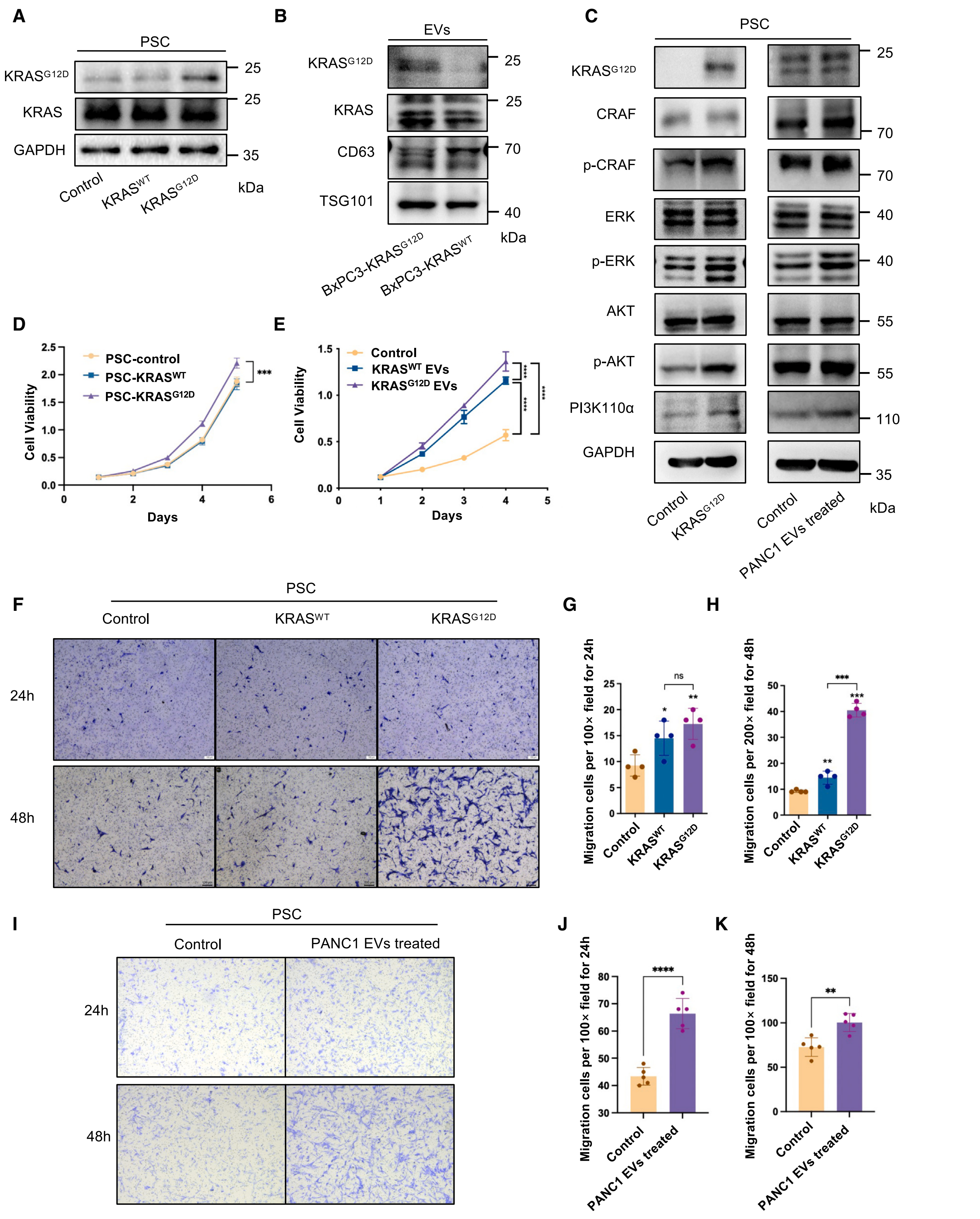

(legend on next page)

of PSC-KRAS^{G12D} (Figures 4A and 4B). RNA sequencing was conducted on both PSC-control and PSC-KRAS^{G12D} samples. KEGG pathway analysis revealed significant upregulation of pathways associated with tumor matrix components, including tight junctions, adherens junctions, and focal adhesions, in PSC-KRAS^{G12D} cells (Figure S2C). These findings indicate that KRAS^{G12D} mutation may promote collagen deposition.

A substantial body of evidence has demonstrated a correlation between a thickened matrix and tumor resistance to chemotherapy.[27,28] Thus, it can be postulated that the thickening of the matrix induced by PSCs harboring the KRAS^{G12D} protein within the tumor microenvironment may contribute to chemotherapy resistance in PDAC. The objective of this study was to investigate the effect of PSCs following the uptake of KRAS^{G12D} protein on chemotherapeutic efficacy. A co-culture model of cancer cells and PSCs with or without gemcitabine stimulation was established. The presence of gemcitabine resulted in enhanced proliferation of BxPC3 cells when co-cultured with PSC-KRAS^{G12D} compared with the PSC-control group. Conversely, the apoptotic rate of cancer cells was significantly reduced in PSC-KRAS^{G12D} co-culture. In the absence of gemcitabine, both active proliferation and apoptosis contributed to a stable fraction of the total cancer cells in both PSC-control and PSC-KRAS^{G12D} groups (Figures 4C and 4D).

Subsequently, *in vivo* experiments were conducted in which a mixture of cancer cells and PSCs was subcutaneously injected into the flanks of NCG mice. Subsequently, the mice were treated with either gemcitabine or phosphate-buffered saline (PBS) every 3 days (Figure 4E). The administration of gemcitabine resulted in a notable reduction in tumor proliferation when BxPC3 cells were combined with the PSC-control compared with the non-gemcitabine-treated group. However, no significant difference in tumor growth was observed between the gemcitabine-treated and control groups for mixtures containing BxPC3 and PSC-KRAS^{G12D} (Figure 4F). Similar results were observed in xenograft models comprising PANC1 cells and PSCs (Figure S2D). Syngeneic models comprising wild-type KRAS Panc02 cells and either 3T3-KRAS^{G12D} or 3T3-control fibroblasts orthotopically injected into the pancreatic tissues of C57BL/6 mice yielded comparable results (Figures 4H, 4I, and S3A). Picrosirius red staining demonstrated augmented collagen deposition in tumors comprising a combination of tumor cells and PSC-KRAS^{G12D}/3T3-KRAS^{G12D} cells compared with those comprising tumor cells and control fibroblasts. Moreover, gemcitabine treatment resulted in augmented collagen infiltration within tumors comprising PSC-control or 3T3-control cells, in accordance with established concepts, indicating that collagen progressively infiltrated the tumor matrix to fill voids created by decreased cellular remnants following chemotherapy (Figures 4G–4J and S2E). Furthermore, gemcitabine treatment resulted in an increase in microvessel density within the tumor matrix, as evidenced by elevated CD31 positivity (Figure S2E). However, alpha smooth muscle actin (αSMA) levels remained stable across all four experimental groups (Figure S2E).

Flow cytometry analysis was conducted to assess the impact of KRAS^{G12D} protein expression in fibroblasts on the immune composition of tumor tissue. These findings indicated that KRAS^{G12D} had a negligible influence on the overall immune microenvironment of the tumor and did not result in notable alterations in the proportion of T and myeloid cells in pancreatic tumors. Moreover, no significant alterations in the immune cell composition were observed between the two groups of tumors subjected to continuous gemcitabine treatment (Figures S3B–S3D).

These findings indicate that CAFs with KRAS^{G12D} may contribute to chemoresistance through augmented collagen deposition and matrix thickening, while exerting a limited influence on the immune microenvironment.

Inhibition of EV secretion effectively reverses the collagen deposition and tumor chemoresistance

Given its established capacity to attenuate the therapeutic efficacy of gemcitabine at the molecular level, we sought to determine whether the encapsulated KRAS protein could exert a comparable effect. Therefore, we used shRNA-mediated knockdown of Rab27a expression to inhibit EV secretion. Rab27a plays a pivotal role in the docking of multivesicular endosomes to the plasma membrane.[29] The modulation of EVs release through manipulation of Rab27a expression has been corroborated in several studies.[29–31] The efficacy of the knockdown was confirmed by western blot analysis in PANC1 and KPC cells (Figure 5A), and by quantifying EV particle numbers using NTA in PANC1 Rab27a_sh1 cells (Figure 5B). The absence of Rab27a did not affect tumor proliferation *in vitro* or *in vivo* (Figures 5C and S4A–S4C). When co-cultured with PSCs, no significant difference was observed in the proportion of apoptotic cells between the PANC1-Rab27a_sh1 and PANC1-control groups in the absence of gemcitabine stimulation. However, after gemcitabine stimulation at an IC_{50} equivalent concentration (4 μM), the

Figure 3. PSCs harboring KRAS^{G12D} protein activate KRAS-related signaling pathways while promoting proliferation and motility during activation
(A) Western blot analysis of KRAS^{G12D} and KRAS proteins in PSC-transfected KRAS^{WT} and KRAS^{G12D} plasmids. (B) Western blot analysis of identified markers and KRAS^{G12D} and KRAS proteins in EVs derived from BxPC3-KRAS^{WT} and BxPC3-KRAS^{G12D} cells. (C) Western blot analysis of KRAS downstream proteins, including c-RAF, p-c-RAF, AKT, p-AKT, ERK, p-ERK, and PI3K110α in PSC-KRAS^{G12D}/PANC1 EV-treated PSCs and control cells. (D) Viability of PSCs overexpressing KRAS^{WT} and KRAS^{G12D} ($n = 5$, $^{***}p < 0.001$, two-way analysis of variance [ANOVA], multiple comparisons). (E) Viability of PSCs treated with KRAS^{WT} and KRAS^{G12D} EVs derived from BxPC3 cells overexpressing KRAS^{WT} and KRAS^{G12D} ($n = 5$, $^{***}p < 0.001$, $^{****}p < 0.0001$, two-way ANOVA, multiple comparisons). (F–H) Representative images and statistical results of cell migration assay of PSC-KRAS^{WT}, PSC-KRAS^{G12D}, and control cells at 24 and 48 h, respectively ($n = 4$, $^{*}p < 0.05$, $^{**}p < 0.01$, $^{***}p < 0.001$, two-tailed t test). (I–K) Representative images and statistical results of the cell migration assay of PSCs treated with EVs derived from PANC1 cells for 24 and 48 h, respectively ($n = 5$, $^{**}p < 0.01$, $^{****}p < 0.0001$, two-tailed t test).

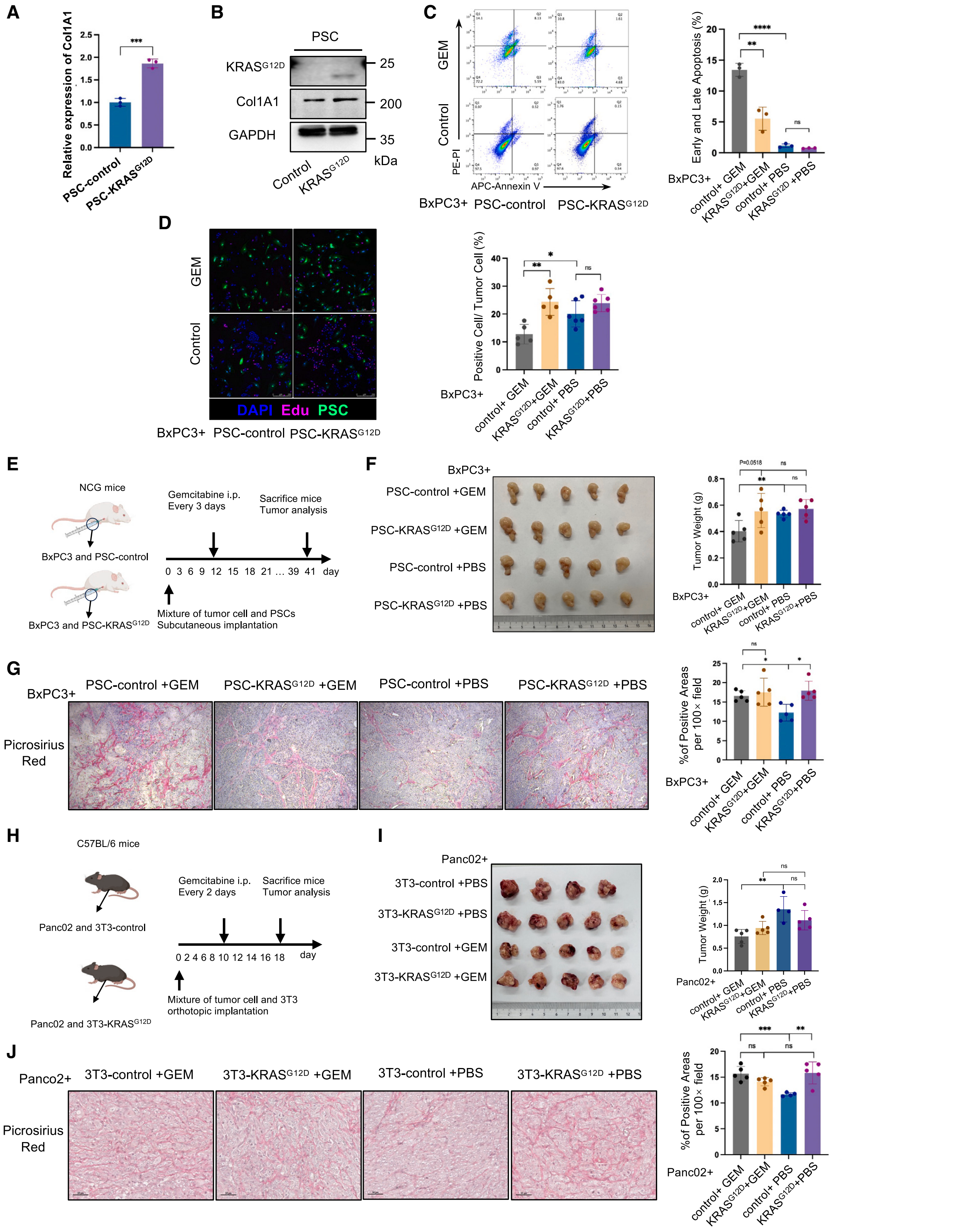

(legend on next page)

PANC1-Rab27a_sh1 group exhibited a greater number of apoptotic cells than the PANC1-control group (Figures 5D and S4D).

Subsequently, a mixture of PSCs and either PANC1-control or PANC1-Rab27a_sh1 cells was subcutaneously injected into NCG mice, with regular administration of gemcitabine or PBS every 3 days (Figure 5E). Following treatment with gemcitabine, tumors derived from both PANC1-control and Rab27a_sh1 mixtures exhibited a moderate reduction in size (Figure 5F). The PANC1-Rab27a_sh1 group exhibited a greater reduction in tumor weight than the PANC1-control group, with reductions of 62% and 41%, respectively (Figure 5G). It is noteworthy that the quantity of $KRAS^{G12D}$ present in PSCs was lower when EV release from cancer cells was inhibited compared with that observed in control cancer cells (Figure 5H). Furthermore, a significant reduction in collagen deposition was observed in the tumors of the PANC1-Rab27a_sh1 group compared with that in the PANC1-control group. This suggests that PSCs with elevated levels of mutant KRAS protein promote collagen deposition within the tumor microenvironment. In addition, gemcitabine treatment increased the proportion of collagen in both the PANC1-control and PANC1-Rab27a_sh1 groups (Figure 5I).

Subsequent investigations were conducted on immunocompetent mice. A mixture of KPC with Rab27a knockdown and 3T3 cells was orthotopically injected into the pancreas of C57BL/6 mice, followed by gemcitabine treatment (Figure 5J). Inhibition of EV release enhanced tumor chemosensitivity and resulted in tumor reduction, whereas the collagen content within the tumor tissue decreased, accompanied by a reduction in Ly6G+ neutrophils and an increase of F4/80+ macrophages within the tumor microenvironment. Furthermore, gemcitabine treatment led to a reduction in T cells, suggesting its potential cytotoxic effect on T cells (Figures 5K–5M, S5A, and S5B). These findings collectively suggest that inhibition of EV delivery fosters the development of a chemosensitive tumor microenvironment, which may be associated with a reduction in collagen deposition.

Specific inhibition of $KRAS^{G12D}$ protein in CAFs results in the reversal of collagen deposition and tumor chemoresistance.

The specific inhibitor, MRTX1133, which targets the $KRAS^{G12D}$ mutation,[16] was used in a co-culture system comprising BxPC3 and PSC-$KRAS^{G12D}$ cells. This was done to ascertain whether the function of the $KRAS^{G12D}$ mutation in PSCs could be inhibited to reverse chemoresistance in PDAC. The IC_{50} values for PSC-$KRAS^{G12D}$ and BxPC3 were determined to be 7.38 and 29.81 μM, respectively (Figure 6A). *In vitro* experiments were conducted by treating a mixture of BxPC3 and PSC-$KRAS^{G12D}$ cells with gemcitabine (4 μM) or MRTX1133 (10 μM). The final concentration of MRTX1133 was selected based on its IC_{50} value, with the objective of selectively inhibiting PSC-$KRAS^{G12D}$ without affecting BxPC3 proliferation. Combination treatment resulted in more apoptosis than gemcitabine monotherapy. Concurrently, MRTX1133 treatment demonstrated effects comparable with those observed in the non-stimulated group with minimal apoptosis (Figures 6B and 6C). *In vivo* studies entailed subcutaneous injection of a mixture of BxPC3 and PSC-$KRAS^{G12D}$ into the right flank of NCG mice, followed by regular administration of gemcitabine and MRTX1133 every 3 days (Figure 6D). The combined treatment demonstrated superior therapeutic efficacy in terms of tumor weight reduction compared with single-agent MRTX1133. However, tumors treated solely with gemcitabine did not exhibit a significant reduction compared with the controls (Figures 6E and 6F). Tumors comprising both BxPC3 cells and PSCs, which lack an effective target for $KRAS^{G12D}$ inhibition, exhibited no discernible differences between gemcitabine alone and combination therapy with gemcitabine and MRTX1133. This finding highlights that MRTX1133 selectively targeted the $KRAS^{G12D}$ mutation (Figures S6A and S6B). Moreover, picrosirius red staining demonstrated that single-agent MRTX1133 treatment resulted in diminished collagen deposition compared with controls, whereas gemcitabine exposure facilitated accelerated collagen accumulation within tumors under concurrent MRTX1133 treatment (Figures 6G and 6H). Given these findings, we put forward the hypothesis that the selective inhibition of $KRAS^{G12D}$ using MRTX1133 may contribute to the reversal of tumor chemoresistance by reducing collagen deposition.

Patients carrying $KRAS^{G12D}$ mutation indicate poor response to conventional treatment

To further substantiate this hypothesis using clinical samples, data were collected from 74 patients diagnosed with PDAC who

Figure 4. $KRAS^{G12D}$-transfected PSCs enhance tumor chemoresistance and facilitate collagen deposition

(A) Quantitative real-time PCR analysis of Col1A1 expression in PSC-$KRAS^{G12D}$ and control cells (n = 3, ***$p < 0.001$, two-tailed t test). (B) Western blot analysis of Col1A1 expression in PSC-$KRAS^{G12D}$ and control cells. (C) Representative images of apoptosis assay showing gemcitabine-treated BxPC3 co-culture with PSC-$KRAS^{G12D}$ or PSC-control cells for 48 h (4 μM) and statistical results of the percentage of early and late apoptotic cells (n = 3, **$p < 0.01$, ****$p < 0.0001$; ns, not significant, two-tailed t test). (D) Representative images of EDU assay of BxPC3 co-culture with PSC-$KRAS^{G12D}$ or PSC-control cells after gemcitabine treatment for 48 h (4 μM) and statistical results of percentage of positive cells accounted for tumor cells. Scale bars, 50 μm (100×). White arrow: $KRAS^{G12D}$-positive CAF (n = 5, *$p < 0.05$, **$p < 0.01$; ns, not significant, two-tailed t test). (E) Schematic protocols of the mixture of BxPC3 and PSC-control or PSC-$KRAS^{G12D}$ subcutaneously injected into the flank of NCG mice with gemcitabine (10 mg/kg) or PBS treatment every 3 days. (F) Representative images and weights of tumors harvested from NCG on day 41 (n = 5, **$p < 0.01$; ns, not significant, two-tailed t test). (G) Statistical results and representative images of picrosirius red staining of tumors for collagen in each group in (D) (n = 5, three fields of view were selected for each tumor tissue, *$p < 0.05$; ns, not significant, two-tailed t test). Scale bars, 50 μm (100×). (H) Schematic protocols of the mixture of Panc02 and 3T3-control or 3T3-$KRAS^{G12D}$ orthotopically injected into the pancreas of C57BL/6 mice with gemcitabine (20 mg/kg) or PBS treatment every 2 days. (I) Representative images and weight of tumors harvested from C57BL/6 mice on day 16 (n = 4 for Panc02- and 3T3 PBS-treated group and n = 5 for other groups, **$p < 0.01$; ns, not significant, two-tailed t test). (J) Statistical results and representative images of picrosirius red staining of tumor for collagen in each group in (G) (n = 4 for Panc02- and 3T3 PBS-treated group and n = 5 for other groups, three fields of view were selected for each tumor tissue, *$p < 0.05$; ns, not significant, two-tailed t test). Scale bars, 50 μm (100×).

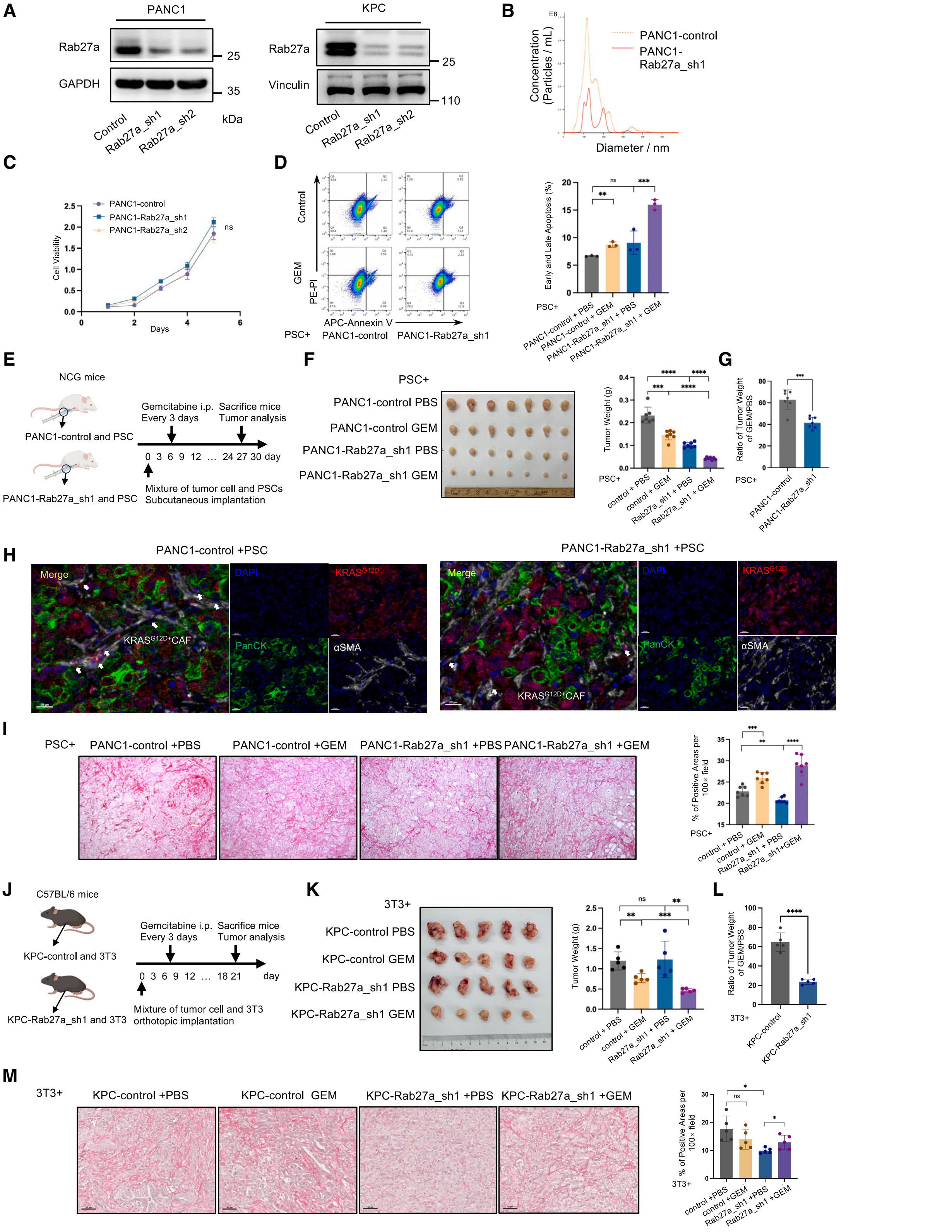

(legend on next page)

underwent chemotherapy at The First Affiliated Hospital of the Zhejiang University School of Medicine. The cohort included 59 patients with KRAS^{G12D} mutations and 15 patients without KRAS mutations. The presence of the KRAS^{G12D} mutation was correlated with adverse outcomes, characterized by a significantly reduced progression-free survival (PFS) duration compared with the non-KRAS mutation cohort during chemotherapy. Specifically, the median survival for the KRAS^{G12D} subgroup was 8.40 months, in contrast to 17.60 months for those with wild-type KRAS (p < 0.05) (Figure 7A). No significant association was observed between KRAS^{G12D} mutation status and tumor size, TNM stage, or other clinical parameters in the pancreatic cancer patient cohort (Table S1). A subset of patients may choose alternative treatment regimens because their current therapy results in tumor progression or if the adverse effects become excessively burdensome. A total of 33 patients with KRAS^{G12D} mutations and 13 patients without such mutations received gemcitabine-based chemotherapy regimens. It is noteworthy that the PFS duration was significantly shorter in patients with a KRAS^{G12D} mutation than in those without a KRAS mutation (Figure 7B). Moreover, no significant differences were observed in the prognostic value of the modified FOLFIRINOX-containing chemotherapy regimens between patients stratified according to KRAS status (Figure S7A). Furthermore, an analysis of survival data from patients with PDAC in the TCGA database indicated that individuals with the KRAS^{G12D} mutation exhibited reduced overall survival following active treatment compared with those with no KRAS mutations (Figure 7C). Consequently, whereas there may be a correlation between increased malignancy and patient prognosis among individuals bearing the KRAS^{G12D} mutation, our findings suggest that patients with PDAC carrying the KRAS^{G12D} mutation exhibit increased resistance to gemcitabine-based chemotherapy.

DISCUSSION

KRAS mutation is regarded as the primary initiating mutation in PDAC, with KRAS^{G12D} mutation representing one of the most frequently occurring forms.[32] Most current research on KRAS^{G12D} in PDAC has focused on tumor cells, where these mutations have been observed to enhance multiple downstream signaling pathways, promote cell proliferation, and induce glucose metabolism, endocytosis, and autophagy. This ultimately leads to tumor progression, metastasis, and chemotherapy resistance.[20,33,34] Tumor cells with mutant KRAS proteins also regulate stromal cells through paracrine secretion, which affects their biological function.[35] CAFs are regulated by secreted factors, including TGF-β and those related to the hedgehog pathway, which originate from these tumor cells.[36,37] Nevertheless, there is a paucity of research on the direct functional implications of KRAS mutations in stromal cells. Previous studies have demonstrated that KRAS^{G12D} protein can be transferred to macrophages via EVs, promoting macrophage polarization toward an M2 phenotype.[21] This study yielded a novel finding: tumor-derived KRAS^{G12D} protein was also detected in CAFs. Following exposure to phenotypic induction cues from neighboring cancer cells,[38] CAFs undergo activation, exhibiting enhanced proliferation and migration, and increased resistance to chemotherapy. This finding further supports the hypothesis that CAFs are educated by mutant KRAS-expressing tumor cells as part of a reciprocal interaction between cancerous cells and surrounding stromal cells. Given these findings, it would be worthwhile to investigate whether other proto-oncogene proteins are transported via EVs released by tumor cells with a similar impact on adjacent stromal cell populations (Figure 7D).

Membrane vesicles or EVs are secreted by a variety of cell types, including tumor cells, CAFs, and macrophages. EVs display considerable heterogeneity in their nucleic acid and protein contents. The presence of EVs carrying KRAS^{G12D} protein has been documented in PDAC. The release of EVs containing KRAS is facilitated by autophagy-dependent ferroptosis in cancer cells.[21] However, in contrast to previous research, we conducted a mass spectrometry analysis of EVs isolated from patient tumors. Despite our inability to identify the specific mutation types, KRAS proteins were present in EVs (Figure 2G). Furthermore, KRAS^{G12D} protein was identified in the CAFs of tumor specimens obtained from patients with this specific mutation (Figure 1A). Given our findings, we put forward the hypothesis that the secretion of KRAS-containing EVs is not confined to a specific microenvironment, but occurs widely within organisms. Several studies have indicated the presence of KRAS mutations in circulating tumor DNA, which may serve as potential markers for liquid biopsy-based

Figure 5. Blocking the secretion of EVs through Rab27a knockdown increases chemosensitivity and reduces collagen deposition

(A) Western blot analysis of Rab27a expression in PANC1-Rab27a_sh1, PANC1-Rab27a_sh2, control, KPC-Rab27a_sh1, KPC-Rab27a_sh2, and control cells. (B) Nanosight analysis of EVs secreted by PANC1-Rab27a_sh1 cells and control cells. (C) Viability of PANC1-Rab27a_sh1, Rab27a_sh2, and control cells (n = 4; ns, not significant, two-tailed t test). (D) Representative images and statistical results of the apoptosis assay in gemcitabine-treated PSCs co-culture with PANC1-Rab27a_sh1 or control cells for 48 h (4 μM) (n = 3, **p < 0.01; ns, not significant, two-tailed t test). (E) Schematic protocols of the mixture of PSCs and PANC1-Rab27a_sh1 and control cells subcutaneously injected into the flank of NCG mice administered gemcitabine (20 mg/kg) every 3 days. (F) Representative images and statistical results of tumors harvested from NCG mice on day 30 (n = 7, ***p < 0.001, ****p < 0.0001, two-tailed t test). (G) Statistical ratio of tumor weight of gemcitabine-treated group and PBS group between PANC1-control and PANC1-Rab27a_sh1 cells co-injected with PSCs (***p < 0.001, two-tailed t test). (H) Representative images of mIHC staining with DAPI, αSMA, PanCK, and KRAS^{G12D} in tumor mixing PANC1 control or PANC1-Rab27a_sh1 cells with PSCs. Scales bars, 20 μm (400×). White arrow: KRAS^{G12D}-positive CAF. (I) Representative images and statistical analysis of picrosirius red staining for collagen in each group in (G) (n = 7, three fields of view were selected for each tumor tissue, **p < 0.01, ***p < 0.001, ****p < 0.0001, two-tailed t test). Scale bars, 50 μm (100×). (J) Schematic protocols of the mixture of 3T3 and KPC-Rab27a_sh1 and control cells orthotopically injected into the pancreas of C57BL/6 mice administered with or without gemcitabine (20 mg/kg) every 3 days. (K) Representative images and statistical results of tumors harvested from C57BL/6 mice on day 21 (n = 5, **p < 0.01, ***p < 0.001; ns, not significant, two-tailed t test). (L) Statistical ratio of tumor weight of gemcitabine-treated group and PBS group between KPC-control and KPC-Rab27a_sh1 cells co-injected with 3T3 (****p < 0.0001, two-tailed t test). (M) Representative images and statistical analysis of picrosirius red staining for collagen in each group in (K) (n = 5, three fields of view were selected for each tumor tissue, *p < 0.05; ns, not significant, two-tailed t test). Scale bars, 50 μm (100×).

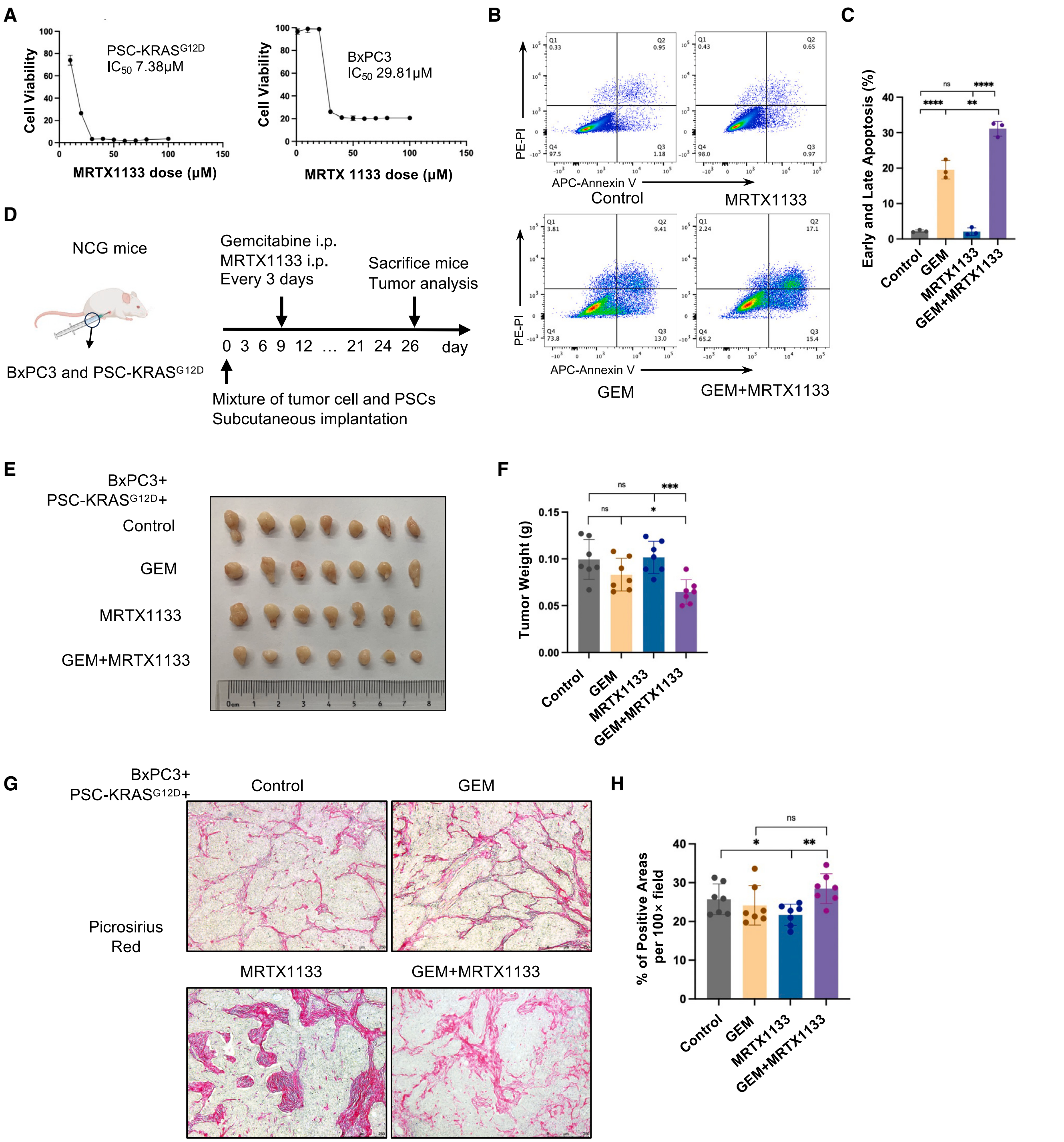

Figure 6. MRTX1133 reverses KRAS^{G12D}-induced tumor chemoresistance and collagen deposition

(A) IC_{50} analysis of PSC-KRAS^{G12D} and BxPC3 cells treated with MRTX1133 for 48 h. (B and C) Representative images and statistical results of apoptosis assay in which the mixture of PSC-KRAS^{G12D} and BxPC3 was stimulated by MRTX1133 (10 μM), gemcitabine (4 μM), and combined treatment for 48 h ($n = 3$, $**p < 0.01$, $****p < 0.0001$; ns, not significant, two-tailed t test). (D) Schematic protocols of the mixture of BxPC3 and PSC-KRAS^{G12D} subcutaneously injected into the flank of NCG mice administered gemcitabine (20 mg/kg), MRTX1133 (10 mg/kg), or their combination every 3 days. (E) Representative images of tumors harvested from NCG mice ($n = 7$). (F) Tumor weight in each group is shown in (E) ($n = 7$, $*p < 0.05$, $***p < 0.001$; ns, not significant, two-tailed t test). (G and H) Representative picrosirius red staining images and further quantification of tumor-infiltrating collagen in each group in (E) ($n = 7$, three fields of view were selected for each tumor tissue, $*p < 0.05$, $**p < 0.01$, two-tailed t test). Scale bars, 50 μm (100×).

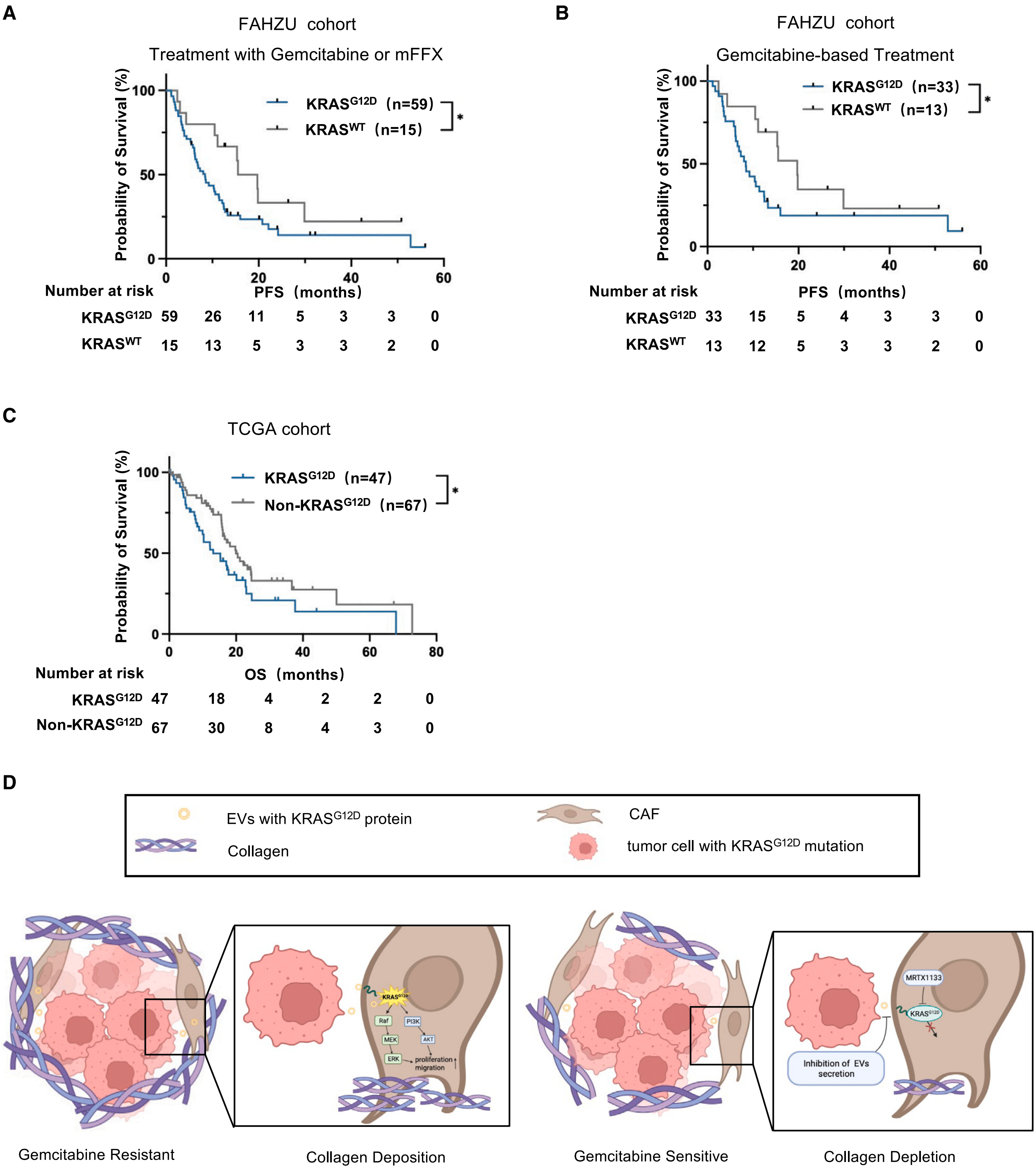

Figure 7. PDAC patients with KRAS^{G12D} mutation are indicated to be less effective to systemic treatment

(A) Survival curve of progression-free survival of patients with KRAS^{G12D} mutation (n = 59) and KRAS^{WT} mutation (n = 15) (*$p < 0.05$, Kaplan-Meier method and Gehan-Breslow-Wilcoxon test). (B) Progression-free survival curve of patients with KRAS^{G12D} mutation (n = 33) and KRAS^{WT} mutation (n = 13) who were treated with gemcitabine (*$p < 0.05$, Kaplan-Meier method and Gehan-Breslow-Wilcoxon test). (C) Survival curve of overall survival of patients with KRAS^{G12D} mutation (n = 47) and non-KRAS^{G12D} mutation (n = 67) in TCGA dataset (*$p < 0.05$, Kaplan-Meier method and Gehan-Breslow-Wilcoxon test). (D) A schematic model displaying tumor chemoresistance with collagen deposition induced by EVs containing KRAS^{G12D} protein delivered between tumor cells and CAFs. Inhibition of EV delivery and specific inhibition of KRAS^{G12D} protein can reverse tumor chemoresistance and collagen reduction.

diagnosis.[39,40] It seems reasonable to posit that KRAS protein may also be present in peripheral blood EVs. Therefore, an exosomal assay for mutant KRAS protein may be a promising novel marker for liquid biopsies.

A substantial number of CAFs and other stromal components are present in pancreatic cancer.[41] The precise role of CAFs in pancreatic cancer progression remains unclear. However, existing evidence suggests that CAFs may serve as both immune suppressors and a physical barrier. Within the tumor microenvironment, CAFs inhibit immune responses, whereas their collagen production and stromal deposition create physical obstacles that impede drug penetration, reducing drug efficacy.[6,7,10,42] In tumors, CAFs and tumor cells mutually influence each other through direct contact or paracrine signaling channels.[42–44] The KRAS proto-oncogene is mutated in pancreatic cancer, leading to rapid proliferation of tumor cells and the onset of oncogenesis. In genetically engineered mouse models of pancreatic cancer, the $KRAS^{G12D}$ mutation in pancreatic acinar cells has been demonstrated to be sufficient to initiate the development of PanIN lesions, which subsequently progress to malignant tumors. The aim of this study was to investigate whether ingestion of $KRAS^{G12D}$ induces tumorigenic transformation of CAFs. It was observed that, upon harboring the $KRAS^{G12D}$ mutation in tumor cells, CAFs underwent phenotypic changes associated with tumor cells, such as enhanced proliferation, migration, and angiogenesis. However, when injected alone into nude mouse models, CAFs did not form tumors, indicating that the acquisition of mutant KRAS proteins alone was insufficient to confer invasive and proliferative abilities similar to those exhibited by the tumor cells.

The development of chemoresistance in pancreatic cancer is a substantial clinical challenge. A substantial body of research has been dedicated to investigating CAF-related chemoresistance. Our study offers novel insights, suggesting that the resistance induced by CAFs may be associated with the acquisition of proto-oncogenes, which leads to collagen deposition. It is widely accepted that the chemoresistance induced by CAFs results from biochemical signaling between tumor cells and CAFs, the secretion of collagen-forming matrix by CAFs, and inadequate vascular perfusion within the tumor.[43,45–47] The presence of the $KRAS^{G12D}$ protein in CAFs promotes collagen deposition, which results in tumor chemoresistance. Previous studies have demonstrated that direct elimination of CAFs can harm the efficacy of chemotherapy, underscoring the necessity of remodeling the stroma and immune microenvironment to mitigate CAF-induced chemoresistance.[48] Given our findings, it may be posited that targeting stromal deposition caused by the uptake of proto-oncogenes in CAFs may enhance the sensitivity of tumors to gemcitabine-based chemotherapy.

In this study, we observed that the chemoresistant phenotype induced by the uptake of $KRAS^{G12D}$ by CAFs could be reversed by the $KRAS^{G12D}$-specific inhibitor MRTX1133. The development of inhibitors targeting KRAS mutations is an ongoing research.[49–51] MRTX1133 is a selective inhibitor specifically designed to target $KRAS^{G12D}$. It has overcome the challenge of targeting this mutation, exhibiting potent efficacy in inhibiting tumor progression driven by this mutation.[16,52] Using animal models of pancreatic cancer, Mahadevan et al. demonstrated the effectiveness of MRTX1133 and reported survival benefits in spontaneously tumorigenic mice. This direct inhibitor of $KRAS^{G12D}$ can induce FAS expression in tumor cells, enhancing the ability of $CD8^+$ T cells to kill them.[53] This study demonstrated that, by targeting $KRAS^{G12D}$ protein in CAFs, MRTX1133 reduced stromal content and enhanced chemosensitivity in pancreatic cancer, providing a novel potential mechanism for MRTX1133-induced tumor regression.

This study has limitations. First, we did not comprehensively investigate the precise molecular mechanism by which CAFs induce chemoresistance following the uptake of $KRAS^{G12D}$ protein. Second, *in vivo* models such as CD63/CD9 Cre-driven lineage-traced mice were not used to further demonstrate EV-mediated transfer of KRAS protein. Third, quantification of collagen in tumors using tissue staining is an inadequate and inaccurate method. Furthermore, the limited availability of tissue samples precluded the evaluation of the relationship between stromal content in patient tumors and their chemotherapeutic responses.

MATERIALS AND METHODS

Cell culture and reagents

PANC1, BxPC3, and Panc02 cell lines were purchased from the American Type Culture Collection (ATCC, Manassas, VA). KPC cell line derived from spontaneous tumors in KrasLSL-G12D, Trp53LSL-R172H, and Pdx1-cre mouse models was kindly provided by Prof. Raghu Kalluri (MD Anderson Cancer Center, Houston, TX). NIH3T3cell line was purchased from Procell (Wuhan, China), PANC1 cells, BxPC3 cells, Panc02 cells, and 3T3 cells were cultured in high-glucose Dulbecco's modified Eagle's medium (DMEM) (Hyclone Cytiva). The KPCs were maintained in McCoy's 5A (modified) medium (Thermo Fisher Scientific, Waltham, MA). Both media were supplemented with 10% fetal bovine serum (FBS) (CE500, Newzerum, New Zealand) and 1% penicillin/streptomycin (CR-15140, Cienry, Zhejiang, China). PSCs were purchased from ScienCell Research Laboratory and cultured in Stellate Cell Conditional Medium (ScienCell Research Laboratory, San Diego, CA). All the cell lines were cultured at 37°C in a humidified atmosphere containing 5% CO_2. The identity of all the cells was verified using short tandem repeat authentication.

Cell transfections

When PANC1, BxPC3 cells, and PSCs reached 30% confluence, they were stably transduced with lentiviral particles encoding human $KRAS^{G12D}$ or the short hairpin RNA Rab27a (Jikai Gene, Shanghai, China). Experiments were performed in accordance with the manufacturer's instructions. After transfection, cells were incubated for 24 h and selected using 2 μg/mL puromycin for 1 week.

HEK293 cells at 70% confluence were transfected with mouse $KRAS^{G12D}$ DNA via the mouse $KRAS^{G12D}$ plasmid using PEI (C0537, Beyotime, Shanghai, China) with psPAX2 and pMD2.G

plasmids (Miaoling, Wuhan, China). The lentivirus was collected and concentrated with PEG8000 (ST483, Beyotime), and NIH3T3cells were transduced with lentivirus in accordance with polybrene (5 μg/mL). After incubation for 24 h, the medium was replaced with fresh medium and the transduced cells were selected after treatment with 2 μg/mL puromycin (InvivoGen, San Diego, CA) for 1 week.

HEK293 cells at 70% confluence were transfected with mouse Rab27a-shRNA DNA via a mouse Rab27a-shRNA plasmid using PEI (C0537, Beyotime) with psPAX2 and pMD2.G plasmids (Miaoling). The lentivirus was collected and concentrated with PEG8000 (ST483, Beyotime), and KPC cells were transduced with lentivirus in accordance with polybrene (5 μg/mL). Following an incubation period of 24 h, the medium was replaced with fresh medium. The transduced cells were then subjected to a 1-week selection process involving treatment with 10 μg/mL puromycin (InvivoGen).

Transfection efficiency was determined by western blotting, and RT-PCR was used to evaluate protein and mRNA expression following cell collection.

Clinical specimen acquisition

Human PDAC tumor tissues were surgically excised and collected from the Department of Hepatobiliary and Pancreatic Surgery of the First Affiliated Hospital of the Zhejiang University School of Medicine (FAHZU). Tissues were formalin-fixed and embedded (FFPE). PDAC samples were obtained from patients who underwent surgical resection. Disease stage was diagnosed according to the National Comprehensive Cancer Network Clinical Practice Guidelines for Pancreatic Adenocarcinoma (version 1.2020). The study was approved by the Ethics Committee of FAHZU, and all patients provided formal consent.

Isolation of primary CAFs

Primary CAFs were isolated from tumors obtained from patients with PDAC. Tumor tissues were minced and digested in RPMI-1640 medium with 2% bovine serum albumin (BSA) (0332 Amresco) and DNase (10 μg/mL) (D5025, Sigma-Aldrich, St. Louis, MO), and collagenase IV (1 mg/mL) (Thermo Fisher Scientific) while shaking at 180 rpm at 37°C for 30 min. The digested tissues were filtered through 70-mm strainers to obtain single cells and centrifuged at 600 × *g* for 5 min at 4°C. Single cells were suspended in RBC lysis buffer for 2 min to remove red blood cells. Cells were cultured in high-glucose DMEM (Hyclone Cytiva) supplemented with 10% FBS and 1% penicillin/streptomycin (CR-15140, Cienry) at 37°C in a humidified atmosphere containing 5% CO_2. The culture medium was changed the following day. The cells were allowed to grow to 80% confluence and were then passaged. The passage was conducted two or three times to obtain CAFs.

Separation of EVs from cell line

EVs were isolated using an Optima XPN-100 ultracentrifuge (Beckman Coulter, CA). PANC1 and BxPC3 cells were seeded in 10-cm dishes. The cell culture medium was replaced with FBS-free and penicillin/streptomycin-free culture medium when the cells reached 70% confluence. After 24 h of culture, the supernatants were collected and referred to as tumor-conditioned medium. The conditioned medium was centrifuged at 300 × *g* for 10 min to remove live cells, 2,000 × *g* for 20 min to remove dead cells, and 12,000 × *g* for 40 min to remove large cell fragments. The supernatants were subjected to ultrafiltration (UFC9003, Millipore, Billerica, MA) at 4,000 × *g* for 40 min. The supernatants were ultracentrifuged at 100,000 × *g* for 70 min. After ultracentrifugation, the sediment was resuspended in a fresh culture for subsequent experiments.

To determine the protein concentration of the EVs, the sediment after ultracentrifugation was resuspended in radioimmunoprecipitation assay (RIPA) lysis buffer (P0013B, Beyotime) and allowed to stand for 30 min on ice. Bicinchoninic acid (BCA) (P0012, Beyotime) was used for quantification. The EVs supernatant was denatured in 4× LDS Sample Buffer (4×) (2785243, Invitrogen, San Diego, CA) and subjected to western blotting analysis.

Separation of EVs from tissue and LC-MS/MS analysis

EVs from the tumors of nine patients with pancreatic cancer and normal tissues were purified and enriched. Tumors were harvested from the visible gray area, and normal tissues were harvested from the pancreatic tissue, 1 cm from the tumor incisal margin. All tissues were reviewed using intraoperative frozen sections and postoperative pathology.

EVs were separated from the tissue using a previously established protocol.[54]

Proteins in the EVs were extracted and quantified, followed by SDS-PAGE and mass spectrometry quality control. The samples were then enzymolyzed and analyzed using mass spectrometry. Raw data were searched for in the database, followed by protein identification and subsequent bioinformatics analysis.

Electron microscopy

Electron microscopy was performed using a transmission electron microscope (JEM-12001×, JEOL, Tokyo, Japan), following standard staining procedures with phosphotungstic acid (Sigma-Aldrich).

Nanosight analysis

Vesicle suspensions with concentrations between 1×10^7/mL and 1×10^9/mL were examined using an NTA nanoparticle tracking analyzer (Nanosight NS300, UK) to determine the size and quantity of isolated particles. A video of 60-s duration was captured at a frame rate of 30 frames/s, and particle movement was analyzed using NTA software (ZetaView 8.02.28).

DNA and cDNA sequencing

DNA and cDNA sequencing were performed by Xiangyin Biotechnology (Hangzhou, China).

Western blot analysis

Proteins were extracted from the cells using RIPA lysis buffer (P0013B, Beyotime) containing a protease inhibitor (P1005, Beyotime). After quantification using BCA (P0012, Beyotime), the protein samples were subjected to SDS-PAGE, followed by transfer onto polyvinylidene fluoride membranes (Millipore). Then, 5% skim milk was used to block nonspecific proteins, and the primary antigen was incubated at 4°C overnight. The membrane was then incubated with horseradish peroxidase-conjugated secondary antibodies at 4°C for 2–4 h. Signals were detected using an enhanced chemiluminescence western blotting substrate kit (Abclonal, Beijing, China). Primary antibody: Ras (G12D Mutant Specific) (14429, Cell Signaling Technology, Boston, MA), KRAS (71835, Cell Signaling Technology), p44/42 MAPK (Erk1/2) (4695, Cell Signaling Technology), phospho-p44/42 MAPK (Erk1/2) (Thr202/Tyr204) (4370, Cell Signaling Technology), phospho-c-Raf (Ser338) (9427, Cell Signaling Technology), RAF1 monoclonal antibody (66592-1-Ig, Proteintech, Wuhan, China), Akt Antibody (9272, Cell Signaling Technology), phospho-Akt (Ser473) antibody (9271, Cell Signaling Technology), PI3 kinase p110α (4249, Cell Signaling Technology), Rab27a (95394, Cell Signaling Technology), GAPDH (AF0006, Beyotime), Col1A1 (72026, Cell Signaling Technology), exosome combination (Calnexin, CD63, Hsp70, TSG101) (ab275018, Abcam, Cambridge, UK).

RNA extraction and quantitative real-time PCR

A quick RNA extraction kit (RN001-50Rxns, Yishan Biotechnology, Shanghai, China) was used for total RNA extraction, followed by determination of the RNA concentration. RNA was reverse transcribed into cDNA using the PrimeScript RT reagent kit (RR047A, Takara, Dalian, China). Quantitative real-time PCR was performed using cDNA as the template in a 10-μL reaction volume on an Applied Biosystems 7500 Fast Real-Time PCR System (Applied Biosystems, Foster City, CA). Relative gene expression was calculated using the standard $2^{-\Delta\Delta Ct}$ method and was normalized to the reference control Actb (encoding β-actin).

Histopathological analysis and IHC staining

The patient samples that underwent histopathological staining were subjected to DNA sequencing to ascertain the prevalence of $KRAS^{G12D}$ in the PDAC samples. The patients included were predominantly individuals with resectable pancreatic cancer who had not undergone neoadjuvant therapy and ranged in age from 18 to 80 years.

Tumor tissues were fixed in 4% neutral formalin, paraffin-embedded, and sectioned at 4 μm thickness. After antigen retrieval and blocking of endogenous peroxidase, the slides were stained with RAS G12D (1:100) (ab221163, Abcam), CD31 (1:200) (77699, Cell Signaling Technology), and αSMA (1:10,000) (ab7817, Abcam). The corresponding secondary antibodies (1:50) (A0208, A0216, Beyotime) were incubated, followed by chromogenic DAB staining (cat. no. GK347011, Gene Tech, Shanghai, China) and enclosed with neutral resin after nuclear staining.

FFPE samples were subjected to picrosirius red staining using a picrosirius red staining kit (24901, Polyscience, Niles, IL).

Representative images of the tumors were captured through the Image Scope software. The quantitative results for IHC staining were obtained using ImageJ software and GraphPad Prism 9 (GraphPad Software, San Diego, CA)

mIHC

The staining was performed using an Opal Polaris 7-Color IHC Kit (Akoya Biosciences, Marlborough, MA) and the process of staining was using a previously established protocol.[55] The primary antibodies were RAS G12D (1:100) (ab221163, Abcam), αSMA (1:1,000) (ab7817, Abcam), and Pan Cytokeratin (1:100) (ab7753, Abcam), incorporating Opal Fluorophores (Opal 690, Opal 520, Opal 570, Opal 620, and Opal 480).

Immunofluorescence staining

The cells were fixed with 4% polyoxymethylene for 10 min and blocked with 3% BSA. The cells were incubated with RAS G12D (1:100) (ab221163, Abcam) at 4°C overnight, followed by incubation with secondary antibody. Fluorescent images of the cells and tissues were acquired using a Dmi8 fluorescence microscope (Leica, Wetzlar, Germany).

Cell viability

Cells were seeded in 96-well plates (Corning, NY) at a density of 2,000 cells per well. For EV-treated PSCs, extracted EVs were mixed with the culture medium. For the IC_{50} assay, cells were treated with gemcitabine hydrochloride (S1149, Selleck, Houston, TX) and MRTX1133 (162369732, Aladdin, Shanghai, China) at different concentrations for 48 h. Optical density was evaluated using a cell counting kit (CCK8, GLPBIO, Montclair, CA), incubated for 2 h at 37°C, and detected at 450 nm using a microplate reader (ELx808, BioTek, Winooski, VT). Cell viability was expressed as a percentage of the specific control and analyzed using GraphPad Prism 9.

Transwell assay

To test cell migration ability, cells were seeded on a 4-μm Transwell plate (Corning) with FBS-free medium, whereas medium with 20% FBS was added to the lower layer. For EV-treated PSCs, the extracted EVs were mixed in an FBS-free medium in the upper layer. All chambers were treated with EVs extracted from 35,000,000 PANC1 cells. The underlying cells were stained with 0.1% crystal violet for a specified period. The migrated cells were imaged using a DMi1 optical microscope (Leica) and analyzed using ImageJ software.

Mice model

NCG mice, BALB/c nude mice, and C57BL/6 mice (all males, 4–6 weeks old) were provided by Ziyuan Experimental Animal Company (Hangzhou, China) and housed under specific pathogen-free conditions in the Experimental Animal Center, First Affiliated Hospital, School of Medicine, Zhejiang University. All animal experiments were approved by the ethics committee of the First Affiliated Hospital,

School of Medicine, Zhejiang University. Animal suffering was minimized to guarantee animal welfare during the study. The animals were randomly assigned to each group. Two independent investigators were blinded to group allocation during data collection and analysis.

A mixture of BxPC3 cells (1×10^6 cells/mouse) and PSC-control or PSC-KRAS^{G12D} cells (5×10^5 cells/mouse) was injected subcutaneously into NCG mice. The tumor size was measured using calipers and recorded. The mice were intraperitoneally injected with gemcitabine (10 mg/kg) or PBS every 3 days 10 times. Tumors were harvested and subjected to IHC staining. The mice were euthanized after 3 weeks of treatment.

A mixture of PANC1 cells (2×10^6 cells/mouse) and PSC-control or PSC-KRAS^{G12D} (1×10^6 cells/mouse) was injected subcutaneously into NCG mice. Mice were intraperitoneally injected with gemcitabine (20 mg/kg) or PBS every 3 days for seven times.

PANC1-control and PANC1-Rab27a_sh1 cells (2×10^6 cells/mouse) were injected subcutaneously into NCG mice. A mixture of PANC1-control or PANC1-Rab27a sh1 cells (2×10^6 cells/mouse) and PSCs (1×10^6 cells/mouse) was injected subcutaneously into NCG mice. The tumor size was measured using calipers and recorded. The mice were intraperitoneally injected with gemcitabine (20 mg/kg) or PBS every 3 days for six times. The mice were sacrificed after 3 weeks of treatment. The tumors were removed and subjected to subsequent experiments.

For combined therapy, a mixture of BxPC3 cells (1×10^6 cells/mouse) and PSC-control or PSC-KRAS^{G12D} (5×10^5 cells/mouse) was injected subcutaneously into NCG mice with gemcitabine (20 mg/kg) (Selleck), MRTX1133 (10 mg/kg) (Aladdin), or combined therapy every t3 days for six times. Mice were euthanized after completing the experiment and tumors were harvested for further study.

A mixture of Panc02 cells (2×10^5 cells/mouse) and 3T3-control or 3T3-KRAS^{G12D} cells (2×10^5 cells/mouse) was injected orthotopically into C57BL/6 mice. Mice were intraperitoneally injected with gemcitabine (20 mg/kg) or PBS every 2 days for four times.

A mixture of 3T3 cells (4×10^5 cells/mouse) and KPC-control or KPC-Rab27a_sh1 cells (4×10^5 cells/mouse) was injected orthotopically into C57BL/6 mice. The mice were intraperitoneally injected with gemcitabine (20 mg/kg) or PBS every 3 days for six times.

Mice were euthanized after completing the experiment and tumors were harvested for further study.

Flow cytometric analysis for tissue samples

The preparation of single-cell suspensions of tissues and the process of tissue staining were carried out using a previously established protocol.[56] The cells were stained on ice for the cell surface epitopes CD45, CD3, CD4, CD8, CD11b, Ly6C, Ly6G, and F4/80 in PBS for 30 min in the dark. Cells were fixed, and permeabilized using a fixation/permeabilization solution kit (555028, BD Biosciences). The cells were also stained for intracellular Perforin and CD206 in permeabilization solution (555028, BD Biosciences). All samples were analyzed by flow cytometry using BD LSRFortessa (BD Biosciences), and the data were analyzed using FlowJo software (BD Biosciences). Antibody: brilliant violet 785 anti-mouse CD45 antibody (103149, BioLegend, San Diego, CA, 1:400), FITC anti-mouse CD3 Antibody (100203, BioLegend), PE/Cy7 anti-mouse CD8a antibody (100722, BioLegend, 1:400), APC/Cy7 anti-mouse CD3 antibody (100329, BioLegend, 1:400), PerCP/Cyanine5.5 anti-mouse/human CD11b antibody (101227, BioLegend, 1:400), brilliant biolet 605 anti-mouse Ly-6C antibody (128035, BioLegend, 1:400), BD Horizon BUV395 rat anti-mouse Ly-6G (563978, BD Biosciences, Franklin Lake, NJ, 1:400), and FITC anti-mouse F4/80 antibody (123108, BioLegend, 1:400), PE anti-mouse perforin antibody (154305, BioLegend, 1:400), and APC anti-mouse CD206 (MMR) antibody (141707, BioLegend, 1:400).

Flow cytometry analysis and CFSE assay

CFSE is widely used in cell proliferation assays and *in vivo* tracking. CFSE can passively diffuse into the cells. Inside the cell, acetate groups are cleaved by intracellular esterases and the molecules are converted to fluorescent esters. EVs extracted from PANC1 cell were stained by CFSE (BioLegend) and treated with PSCs for 12 h. The cells were digested, washed with PBS, and stained with CFSE. Fluorescence intensity was detected in the FITC channel using CytoFLEX (Beckman Coulter), and the data was analyzed using FlowJo software.

Cell apoptosis assay and EDU assay

Tumor cells and PSCs were co-cultured and treated with gemcitabine-HCL (4 μM) or MRTX1133 (10 μM) for 48 h. For the cell apoptosis assay, cells were digested and washed in PBS, stained with PI and Annexin V, and detected by PE and APC channels using a CytoFLEX LX flow cytometer (Beckman Coulter). The data were analyzed using FlowJo software.

For the EDU assay, tumor cells and PSCs were analyzed using an EDU Cell Proliferation Kit (C0081, Beyotime). Fluorescence images of cells were acquired using a TCS SP8 X confocal microscope (Leica).

PKH26 staining

EVs extracted from PANC1 cells were stained with PKH26 (Sigma) and treated with PSCs for 12 h. Fluorescence images of cells were obtained using a Dmi8 fluorescence microscope (Leica).

Statistical analysis

Statistical analyses were performed using Prism software (GraphPad, version 9.0). Data for all experiments from at least three biological replicates are presented as mean ± SD. The significance of the difference between two groups was evaluated using Student's t test, whereas one-way and repeated measures analysis of variance was used to compare multiple groups. Differences in survival curves were analyzed using the Kaplan-Meier method and the

Gehan-Breslow-Wilcoxon test. Statistical significance was set at $p < 0.05$.

DATA AND CODE AVAILABILITY

Figure 7C was generated from publicly available TCGA databases. The images in the schematic figure and Figure 7D were derived from Biorender.

ACKNOWLEDGMENTS

We would like to acknowledge the Animal Experimental Ethical Inspection of FAHZU for their ethical animal care. We would like to acknowledge funding from the Joint Fund for Regional Innovation and Development of National Natural Science Foundation of China (U23A20462), the National Key Research and Development Program (2020YFA0804300), the National Natural Science Foundation of China (82103395, 82071867, 81871925, U20A20378, and 82188102), "Ling Yan" Research and Development Program of Department of Zhejiang Province Science and Technology (2024C03167), Zhejiang Provincial Natural Science Foundation of China (LY23H160023), the Key Research and Development Program of Zhejiang Province (2020C03117), the Fundamental Research Funds for the Zhejiang Provincial Universities (2021XZZX031), and the China Postdoctoral Science Foundation (2020M681887).

AUTHOR CONTRIBUTIONS

T.L. and X.B. supervised the study. J.Y. conceived the study and designed the experiments. X.L. and J.Y. performed most experiments and acquired the data. X.L. and J.Y. conducted data analyses and interpretation. Y.H., S.H., Y.Z., J.W., and Y.W. contributed to technical assistance. Y.H. collected clinical data. X.L. and J.Y. drafted and revised the manuscript. All other authors discussed and commented on the manuscript. X.L. and J.Y. contributed equally to this work, and T.L. and X.B. shared the senior authorship of this study. All authors revised and approved the final version of the manuscript.

DECLARATION OF INTERESTS

The authors declare no competing interests.

SUPPLEMENTAL INFORMATION

Supplemental information can be found online at https://doi.org/10.1016/j.ymthe.2025.01.023.

REFERENCES

1. Halbrook, C.J., Lyssiotis, C.A., Pasca di Magliano, M., and Maitra, A. (2023). Pancreatic cancer: Advances and challenges. Cell *186*, 1729–1754. https://doi.org/10.1016/j.cell.2023.02.014.

2. Von Hoff, D.D., Ervin, T., Arena, F.P., Chiorean, E.G., Infante, J., Moore, M., Seay, T., Tjulandin, S.A., Ma, W.W., Saleh, M.N., et al. (2013). Increased survival in pancreatic cancer with nab-paclitaxel plus gemcitabine. N. Engl. J. Med. *369*, 1691–1703. https://doi.org/10.1056/NEJMoa1304369.

3. Seufferlein, T., Uhl, W., Kornmann, M., Algül, H., Friess, H., König, A., Ghadimi, M., Gallmeier, E., Bartsch, D.K., Lutz, M.P., et al. (2023). Perioperative or only adjuvant gemcitabine plus nab-paclitaxel for resectable pancreatic cancer (NEONAX)-a randomized phase II trial of the AIO pancreatic cancer group. Ann. Oncol. *34*, 91–100. https://doi.org/10.1016/j.annonc.2022.09.161.

4. Klein-Brill, A., Amar-Farkash, S., Lawrence, G., Collisson, E.A., and Aran, D. (2022). Comparison of FOLFIRINOX vs Gemcitabine Plus Nab-Paclitaxel as First-Line Chemotherapy for Metastatic Pancreatic Ductal Adenocarcinoma. JAMA Netw. Open *5*, e2216199. https://doi.org/10.1001/jamanetworkopen.2022.16199.

5. Hosein, A.N., Brekken, R.A., and Maitra, A. (2020). Pancreatic cancer stroma: an update on therapeutic targeting strategies. Nat. Rev. Gastroenterol. Hepatol. *17*, 487–505. https://doi.org/10.1038/s41575-020-0300-1.

6. Huang, H., Wang, Z., Zhang, Y., Pradhan, R.N., Ganguly, D., Chandra, R., Murimwa, G., Wright, S., Gu, X., Maddipati, R., et al. (2022). Mesothelial cell-derived antigen-presenting cancer-associated fibroblasts induce expansion of regulatory T cells in pancreatic cancer. Cancer Cell *40*, 656–673.e7. https://doi.org/10.1016/j.ccell.2022.04.011.

7. Mao, X., Xu, J., Wang, W., Liang, C., Hua, J., Liu, J., Zhang, B., Meng, Q., Yu, X., and Shi, S. (2021). Crosstalk between cancer-associated fibroblasts and immune cells in the tumor microenvironment: new findings and future perspectives. Mol. Cancer *20*, 131. https://doi.org/10.1186/s12943-021-01428-1.

8. Shi, L., Zhu, W., Huang, Y., Zhuo, L., Wang, S., Chen, S., Zhang, B., and Ke, B. (2022). Cancer-associated fibroblast-derived exosomal microRNA-20a suppresses the PTEN/PI3K-AKT pathway to promote the progression and chemoresistance of non-small cell lung cancer. Clin. Transl. Med. *12*, e989. https://doi.org/10.1002/ctm2.989.

9. Ren, J., Ding, L., Zhang, D., Shi, G., Xu, Q., Shen, S., Wang, Y., Wang, T., and Hou, Y. (2018). Carcinoma-associated fibroblasts promote the stemness and chemoresistance of colorectal cancer by transferring exosomal lncRNA H19. Theranostics *8*, 3932–3948. https://doi.org/10.7150/thno.25541.

10. Schuth, S., Le Blanc, S., Krieger, T.G., Jabs, J., Schenk, M., Giese, N.A., Büchler, M.W., Eils, R., Conrad, C., and Strobel, O. (2022). Patient-specific modeling of stroma-mediated chemoresistance of pancreatic cancer using a three-dimensional organoid-fibroblast co-culture system. J. Exp. Clin. Cancer Res. *41*, 312. https://doi.org/10.1186/s13046-022-02519-7.

11. Ohlund, D., Handly-Santana, A., Biffi, G., Elyada, E., Almeida, A.S., Ponz-Sarvise, M., Corbo, V., Oni, T.E., Hearn, S.A., Lee, E.J., et al. (2017). Distinct populations of inflammatory fibroblasts and myofibroblasts in pancreatic cancer. J. Exp. Med. *214*, 579–596. https://doi.org/10.1084/jem.20162024.

12. Omary, M.B., Lugea, A., Lowe, A.W., and Pandol, S.J. (2007). The pancreatic stellate cell: a star on the rise in pancreatic diseases. J. Clin. Invest. *117*, 50–59. https://doi.org/10.1172/JCI30082.

13. Faivre, S., Santoro, A., Kelley, R.K., Gane, E., Costentin, C.E., Gueorguieva, I., Smith, C., Cleverly, A., Lahn, M.M., Raymond, E., et al. (2019). Novel transforming growth factor beta receptor I kinase inhibitor galunisertib (LY2157299) in advanced hepatocellular carcinoma. Liver Int. *39*, 1468–1477. https://doi.org/10.1111/liv.14113.

14. Chauhan, V.P., Martin, J.D., Liu, H., Lacorre, D.A., Jain, S.R., Kozin, S.V., Stylianopoulos, T., Mousa, A.S., Han, X., Adstamongkonkul, P., et al. (2013). Angiotensin inhibition enhances drug delivery and potentiates chemotherapy by decompressing tumour blood vessels. Nat. Commun. *4*, 2516. https://doi.org/10.1038/ncomms3516.

15. Doherty, G.J., Tempero, M., and Corrie, P.G. (2018). HALO-109-301: a Phase III trial of PEGPH20 (with gemcitabine and nab-paclitaxel) in hyaluronic acid-high stage IV pancreatic cancer. Future Oncol. *14*, 13–22. https://doi.org/10.2217/fon-2017-0338.

16. Hallin, J., Bowcut, V., Calinisan, A., Briere, D.M., Hargis, L., Engstrom, L.D., Laguer, J., Medwid, J., Vanderpool, D., Lifset, E., et al. (2022). Anti-tumor efficacy of a potent and selective non-covalent KRAS(G12D) inhibitor. Nat. Med. *28*, 2171–2182. https://doi.org/10.1038/s41591-022-02007-7.

17. Huang, L., Guo, Z., Wang, F., and Fu, L. (2021). KRAS mutation: from undruggable to druggable in cancer. Signal Transduct. Target. Ther. *6*, 386. https://doi.org/10.1038/s41392-021-00780-4.

18. Hunter, J.C., Manandhar, A., Carrasco, M.A., Gurbani, D., Gondi, S., and Westover, K.D. (2015). Biochemical and Structural Analysis of Common Cancer-Associated KRAS Mutations. Mol. Cancer Res. *13*, 1325–1335. https://doi.org/10.1158/1541-7786.Mcr-15-0203.

19. Tape, C.J., Ling, S., Dimitriadi, M., McMahon, K.M., Worboys, J.D., Leong, H.S., Norrie, I.C., Miller, C.J., Poulogiannis, G., Lauffenburger, D.A., and Jørgensen, C. (2016). Oncogenic KRAS Regulates Tumor Cell Signaling via Stromal Reciprocation. Cell *165*, 1818. https://doi.org/10.1016/j.cell.2016.05.079.

20. Dey, P., Li, J., Zhang, J., Chaurasiya, S., Strom, A., Wang, H., Liao, W.T., Cavallaro, F., Denz, P., Bernard, V., et al. (2020). Oncogenic KRAS-Driven Metabolic Reprogramming in Pancreatic Cancer Cells Utilizes Cytokines from the Tumor Microenvironment. Cancer Discov. *10*, 608–625. https://doi.org/10.1158/2159-8290.CD-19-0297.

21. Dai, E., Han, L., Liu, J., Xie, Y., Kroemer, G., Klionsky, D.J., Zeh, H.J., Kang, R., Wang, J., and Tang, D. (2020). Autophagy-dependent ferroptosis drives tumor-associated macrophage polarization via release and uptake of oncogenic KRAS protein. Autophagy *16*, 2069–2083. https://doi.org/10.1080/15548627.2020.1714209.

22. Hayashi, A., Hong, J., and Iacobuzio-Donahue, C.A. (2021). The pancreatic cancer genome revisited. Nat. Rev. Gastroenterol. Hepatol. *18*, 469–481. https://doi.org/10.1038/s41575-021-00463-z.

23. Caligiuri, G., and Tuveson, D.A. (2023). Activated fibroblasts in cancer: Perspectives and challenges. Cancer Cell *41*, 434–449. https://doi.org/10.1016/j.ccell.2023.02.015.

24. Ali, S., Suresh, R., Banerjee, S., Bao, B., Xu, Z., Wilson, J., Philip, P.A., Apte, M., and Sarkar, F.H. (2015). Contribution of microRNAs in understanding the pancreatic tumor microenvironment involving cancer associated stellate and fibroblast cells. Am. J. Cancer Res. *5*, 1251–1264.

25. Heinrich, M.A., Uboldi, I., Kuninty, P.R., Ankone, M.J.K., van Baarlen, J., Zhang, Y.S., Jain, K., and Prakash, J. (2023). Microarchitectural mimicking of stroma-induced vasculature compression in pancreatic tumors using a 3D engineered model. Bioact. Mater. *22*, 18–33. https://doi.org/10.1016/j.bioactmat.2022.09.015.

26. Drosten, M., and Barbacid, M. (2020). Targeting the MAPK Pathway in KRAS-Driven Tumors. Cancer Cell *37*, 543–550. https://doi.org/10.1016/j.ccell.2020.03.013.

27. Yeung, T.L., Leung, C.S., Yip, K.P., Sheng, J., Vien, L., Bover, L.C., Birrer, M.J., Wong, S.T.C., and Mok, S.C. (2019). Anticancer Immunotherapy by MFAP5 Blockade Inhibits Fibrosis and Enhances Chemosensitivity in Ovarian and Pancreatic Cancer. Clin. Cancer Res. *25*, 6417–6428. https://doi.org/10.1158/1078-0432.CCR-19-0187.

28. Peran, I., Dakshanamurthy, S., McCoy, M.D., Mavropoulos, A., Allo, B., Sebastian, A., Hum, N.R., Sprague, S.C., Martin, K.A., Pishvaian, M.J., et al. (2021). Cadherin 11 Promotes Immunosuppression and Extracellular Matrix Deposition to Support Growth of Pancreatic Tumors and Resistance to Gemcitabine in Mice. Gastroenterology *160*, 1359–1372.e13. https://doi.org/10.1053/j.gastro.2020.11.044.

29. Ostrowski, M., Carmo, N.B., Krumeich, S., Fanget, I., Raposo, G., Savina, A., Moita, C.F., Schauer, K., Hume, A.N., Freitas, R.P., et al. (2010). Rab27a and Rab27b control different steps of the exosome secretion pathway. Nat. Cell Biol. *12*, 19–30. https://doi.org/10.1038/ncb2000.

30. Wang, G., Li, J., Bojmar, L., Chen, H., Li, Z., Tobias, G.C., Hu, M., Homan, E.A., Lucotti, S., Zhao, F., et al. (2023). Tumour extracellular vesicles and particles induce liver metabolic dysfunction. Nature *618*, 374–382. https://doi.org/10.1038/s41586-023-06114-4.

31. Poggio, M., Hu, T., Pai, C.C., Chu, B., Belair, C.D., Chang, A., Montabana, E., Lang, U.E., Fu, Q., Fong, L., and Blelloch, R. (2019). Suppression of Exosomal PD-L1 Induces Systemic Anti-tumor Immunity and Memory. Cell *177*, 414–427.e13. https://doi.org/10.1016/j.cell.2019.02.016.

32. Buscail, L., Bournet, B., and Cordelier, P. (2020). Role of oncogenic KRAS in the diagnosis, prognosis and treatment of pancreatic cancer. Nat. Rev. Gastroenterol. Hepatol. *17*, 153–168. https://doi.org/10.1038/s41575-019-0245-4.

33. Jonckheere, N., Vasseur, R., and Van Seuningen, I. (2017). The cornerstone K-RAS mutation in pancreatic adenocarcinoma: From cell signaling network, target genes, biological processes to therapeutic targeting. Crit. Rev. Oncol. Hematol. *111*, 7–19. https://doi.org/10.1016/j.critrevonc.2017.01.002.

34. Zhang, W., Nandakumar, N., Shi, Y., Manzano, M., Smith, A., Graham, G., Gupta, S., Vietsch, E.E., Laughlin, S.Z., Wadhwa, M., et al. (2014). Downstream of mutant KRAS, the transcription regulator YAP is essential for neoplastic progression to pancreatic ductal adenocarcinoma. Sci. Signal. *7*, ra42. https://doi.org/10.1126/scisignal.2005049.

35. Tape, C.J., Ling, S., Dimitriadi, M., McMahon, K.M., Worboys, J.D., Leong, H.S., Norrie, I.C., Miller, C.J., Poulogiannis, G., Lauffenburger, D.A., and Jørgensen, C. (2016). Oncogenic KRAS Regulates Tumor Cell Signaling via Stromal Reciprocation. Cell *165*, 910–920. https://doi.org/10.1016/j.cell.2016.03.029.

36. Fang, Z., Meng, Q., Xu, J., Wang, W., Zhang, B., Liu, J., Liang, C., Hua, J., Zhao, Y., Yu, X., and Shi, S. (2023). Signaling pathways in cancer-associated fibroblasts: recent advances and future perspectives. Cancer Commun. (Lond). *43*, 3–41. https://doi.org/10.1002/cac2.12392.

37. Steele, N.G., Biffi, G., Kemp, S.B., Zhang, Y., Drouillard, D., Syu, L., Hao, Y., Oni, T.E., Brosnan, E., Elyada, E., et al. (2021). Inhibition of Hedgehog Signaling Alters Fibroblast Composition in Pancreatic Cancer. Clin. Cancer Res. *27*, 2023–2037. https://doi.org/10.1158/1078-0432.CCR-20-3715.

38. Li, B., Chen, Y., Liang, L., Wang, Y., Huang, W., Zhao, K., Liu, S., Deng, G., and Chen, J. (2022). Tumor-derived extracellular vesicles shuttle c-Myc to promote gastric cancer growth and metastasis via the KCNQ1OT1/miR-556-3p/CLIC1 axis. Cell Death Dis. *13*, 217. https://doi.org/10.1038/s41419-021-04446-5.

39. Guo, S., Shi, X., Shen, J., Gao, S., Wang, H., Shen, S., Pan, Y., Li, B., Xu, X., Shao, Z., and Jin, G. (2020). Preoperative detection of KRAS G12D mutation in ctDNA is a powerful predictor for early recurrence of resectable PDAC patients. Br. J. Cancer *122*, 857–867. https://doi.org/10.1038/s41416-019-0704-2.

40. Montagut, C., Vidal, J., and Visa, L. (2018). KRAS mutations in ctDNA: a promising new biomarker in advanced pancreatic cancer. Ann. Oncol. *29*, 2280–2282. https://doi.org/10.1093/annonc/mdy484.

41. Whittle, M.C., and Hingorani, S.R. (2019). Fibroblasts in Pancreatic Ductal Adenocarcinoma: Biological Mechanisms and Therapeutic Targets. Gastroenterology *156*, 2085–2096. https://doi.org/10.1053/j.gastro.2018.12.044.

42. Vennin, C., Mélénec, P., Rouet, R., Nobis, M., Cazet, A.S., Murphy, K.J., Herrmann, D., Reed, D.A., Lucas, M.C., Warren, S.C., et al. (2019). CAF hierarchy driven by pancreatic cancer cell p53-status creates a pro-metastatic and chemoresistant environment via perlecan. Nat. Commun. *10*, 3637. https://doi.org/10.1038/s41467-019-10968-6.

43. Feig, C., Jones, J.O., Kraman, M., Wells, R.J.B., Deonarine, A., Chan, D.S., Connell, C.M., Roberts, E.W., Zhao, Q., Caballero, O.L., et al. (2013). Targeting CXCL12 from FAP-expressing carcinoma-associated fibroblasts synergizes with anti-PD-L1 immunotherapy in pancreatic cancer. Proc. Natl. Acad. Sci. USA *110*, 20212–20217. https://doi.org/10.1073/pnas.1320318110.

44. Yuan, M., Tu, B., Li, H., Pang, H., Zhang, N., Fan, M., Bai, J., Wang, W., Shu, Z., DuFort, C.C., et al. (2022). Cancer-associated fibroblasts employ NUFIP1-dependent autophagy to secrete nucleosides and support pancreatic tumor growth. Nat. Cancer *3*, 945–960. https://doi.org/10.1038/s43018-022-00426-6.

45. Bordeleau, F., Mason, B.N., Lollis, E.M., Mazzola, M., Zanotelli, M.R., Somasegar, S., Califano, J.P., Montague, C., LaValley, D.J., Huynh, J., et al. (2017). Matrix stiffening promotes a tumor vasculature phenotype. Proc. Natl. Acad. Sci. USA *114*, 492–497. https://doi.org/10.1073/pnas.1613855114.

46. Saatci, O., Kaymak, A., Raza, U., Ersan, P.G., Akbulut, O., Banister, C.E., Sikirzhytski, V., Tokat, U.M., Aykut, G., Ansari, S.A., et al. (2020). Targeting lysyl oxidase (LOX) overcomes chemotherapy resistance in triple negative breast cancer. Nat. Commun. *11*, 2416. https://doi.org/10.1038/s41467-020-16199-4.

47. Dangi-Garimella, S., Krantz, S.B., Barron, M.R., Shields, M.A., Heiferman, M.J., Grippo, P.J., Bentrem, D.J., and Munshi, H.G. (2011). Three-dimensional collagen I promotes gemcitabine resistance in pancreatic cancer through MT1-MMP-mediated expression of HMGA2. Cancer Res. *71*, 1019–1028. https://doi.org/10.1158/0008-5472.CAN-10-1855.

48. Ozdemir, B.C., Pentcheva-Hoang, T., Carstens, J.L., Zheng, X., Wu, C.C., Simpson, T.R., Laklai, H., Sugimoto, H., Kahlert, C., Novitskiy, S.V., et al. (2014). Depletion of carcinoma-associated fibroblasts and fibrosis induces immunosuppression and accelerates pancreas cancer with reduced survival. Cancer Cell *25*, 719–734. https://doi.org/10.1016/j.ccr.2014.04.005.

49. Bockorny, B., Macarulla, T., Semenisty, V., Borazanci, E., Feliu, J., Ponz-Sarvise, M., Abad, D.G., Oberstein, P., Alistar, A., Muñoz, A., et al. (2021). Motixafortide and Pembrolizumab Combined to Nanoliposomal Irinotecan, Fluorouracil, and Folinic Acid in Metastatic Pancreatic Cancer: The COMBAT/KEYNOTE-202 Trial. Clin. Cancer Res. *27*, 5020–5027. https://doi.org/10.1158/1078-0432.CCR-21-0929.

50. Kim, D., Herdeis, L., Rudolph, D., Zhao, Y., Böttcher, J., Vides, A., Ayala-Santos, C.I., Pourfarjam, Y., Cuevas-Navarro, A., Xue, J.Y., et al. (2023). Pan-KRAS inhibitor disables oncogenic signalling and tumour growth. Nature *619*, 160–166. https://doi.org/10.1038/s41586-023-06123-3.

51. de Langen, A.J., Johnson, M.L., Mazieres, J., Dingemans, A.M.C., Mountzios, G., Pless, M., Wolf, J., Schuler, M., Lena, H., Skoulidis, F., et al. (2023). Sotorasib versus docetaxel for previously treated non-small-cell lung cancer with KRAS(G12C) mutation: a randomised, open-label, phase 3 trial. Lancet *401*, 733–746. https://doi.org/10.1016/S0140-6736(23)00221-0.

52. Kemp, S.B., Cheng, N., Markosyan, N., Sor, R., Kim, I.K., Hallin, J., Shoush, J., Quinones, L., Brown, N.V., Bassett, J.B., et al. (2023). Efficacy of a Small-Molecule Inhibitor of KrasG12D in Immunocompetent Models of Pancreatic Cancer. Cancer Discov. *13*, 298–311. https://doi.org/10.1158/2159-8290.CD-22-1066.

53. Mahadevan, K.K., McAndrews, K.M., LeBleu, V.S., Yang, S., Lyu, H., Li, B., Sockwell, A.M., Kirtley, M.L., Morse, S.J., Moreno Diaz, B.A., et al. (2023). KRAS(G12D) inhibition reprograms the microenvironment of early and advanced pancreatic cancer to promote FAS-mediated killing by CD8(+) T cells. Cancer Cell *41*, 1606–1620.e8. https://doi.org/10.1016/j.ccell.2023.07.002.

54. Vella, L.J., Scicluna, B.J., Cheng, L., Bawden, E.G., Masters, C.L., Ang, C.S., Willamson, N., McLean, C., Barnham, K.J., and Hill, A.F. (2017). A rigorous method to enrich for exosomes from brain tissue. J. Extracell. Vesicles *6*, 1348885. https://doi.org/10.1080/20013078.2017.1348885.

55. Zheng, C., Wang, J., Zhou, Y., Duan, Y., Zheng, R., Xie, Y., Wei, X., Wu, J., Shen, H., Ye, M., et al. (2024). IFNalpha-induced BST2(+) tumor-associated macrophages facilitate immunosuppression and tumor growth in pancreatic cancer by ERK-CXCL7 signaling. Cell Rep. *43*, 114088. https://doi.org/10.1016/j.celrep.2024.114088.

56. Zhang, X., Huang, X., Xu, J., Li, E., Lao, M., Tang, T., Zhang, G., Guo, C., Zhang, X., Chen, W., et al. (2021). NEK2 inhibition triggers anti-pancreatic cancer immunity by targeting PD-L1. Nat. Commun. *12*, 4536. https://doi.org/10.1038/s41467-021-24769-3.

YMTHE, Volume *33*

Supplemental Information

Pancreatic cancer-derived extracellular vesicles enhance chemoresistance by delivering $KRAS^{G12D}$ protein to cancer-associated fibroblasts

Xinyuan Liu, Jiaqi Yang, Sicong Huang, Yifan Hong, Yupeng Zhu, Jianing Wang, Yi Wang, Tingbo Liang, and Xueli Bai

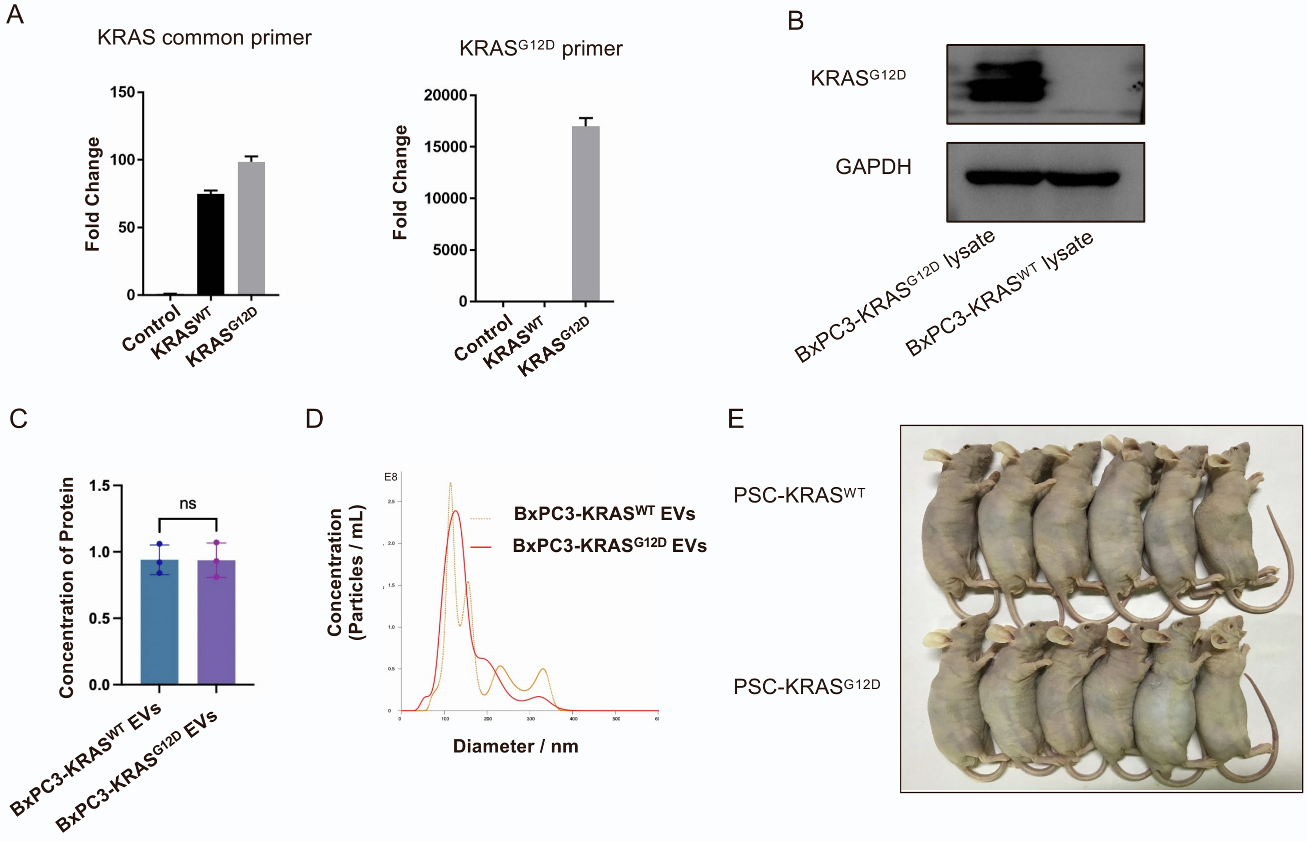

Figure S1. PSCs overexpressing KRAS^{G12D} acquire biological function.

(A) Quantitative Real-time PCR of KRAS^{WT} and KRAS^{G12D} expression in PSC-KRAS^{WT}, PSC-KRAS^{G12D} and control cells (n = 3). (B) Western blot analysis of KRAS^{G12D} proteins in BxPC3-KRAS^{WT} and BxPC3-KRAS^{G12D} cells. (C) BCA analysis of protein concentration of EVs collected from 20,000,000 cells of BxPC3-KRAS^{WT} and BxPC3-KRAS^{G12D} cells (n = 3). (D) Nanosight analysis of EVs concentration collected from 20,000,000 cells of BxPC3-KRAS^{WT} and BxPC3-KRAS^{G12D} cells. (E) Representative images of nude mice bearing PSC-KRAS^{WT} and PSC-KRAS^{G12D} in right flank (n = 6).

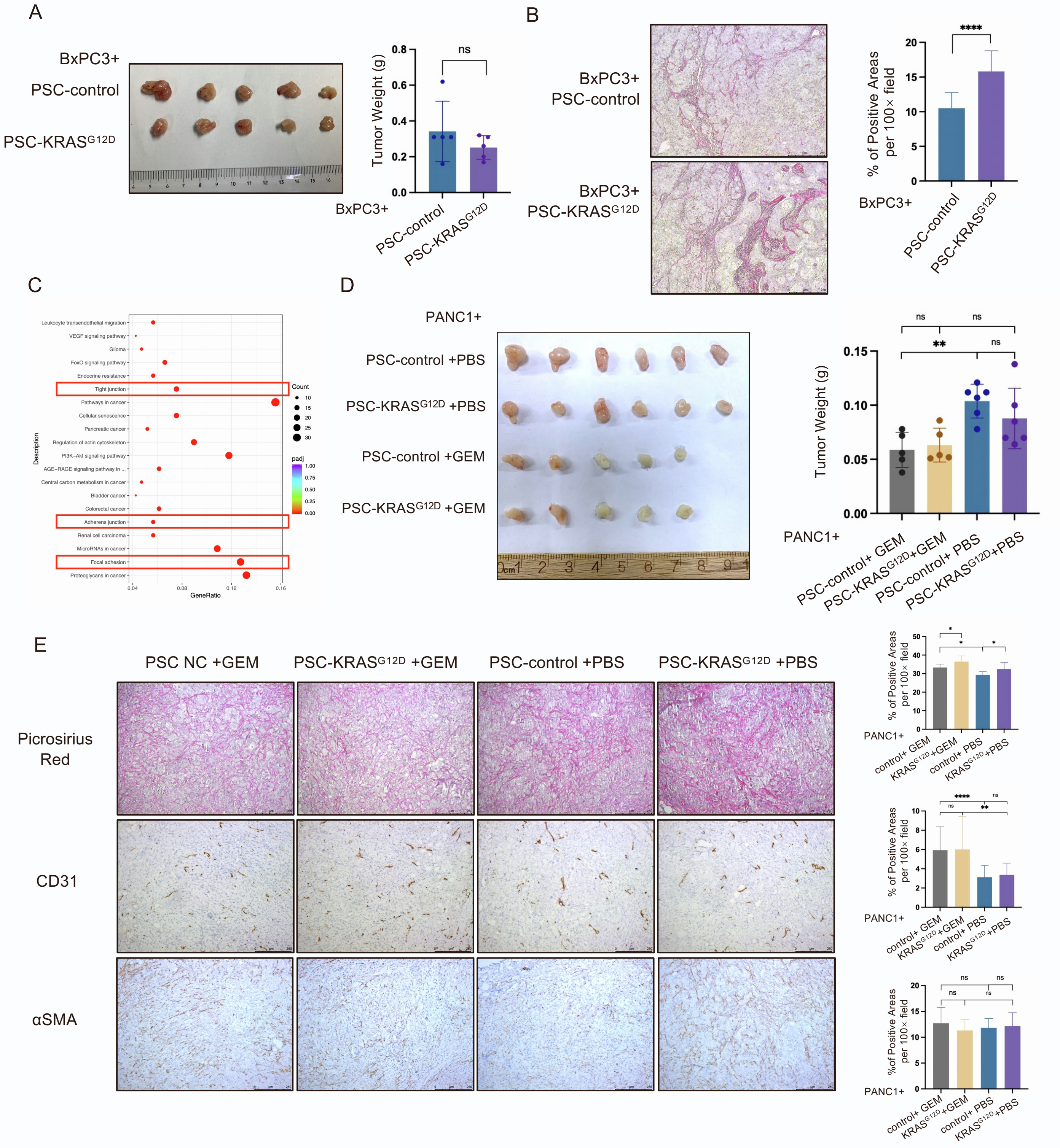

A
BxPC3+
PSC-control
PSC-KRASG12D
Tumor Weight (g)
ns
BxPC3+
PSC-control
PSC-KRASG12D
B
BxPC3+
PSC-control
BxPC3+
PSC-KRASG12D
% of Positive Areas
per 100× field
****
C
Leukocyte transendothelial migration
VEGF signaling pathway
Glioma
FoxO signaling pathway
Endocrine resistance
Tight junction
Pathways in cancer
Cellular senescence
Pancreatic cancer
Regulation of actin cytoskeleton
PI3K−Akt signaling pathway
AGE−RAGE signaling pathway in ...
Central carbon metabolism in cancer
Bladder cancer
Colorectal cancer
Adherens junction
Renal cell carcinoma
MicroRNAs in cancer
Focal adhesion
Proteoglycans in cancer
Description
GeneRatio
Count
padj
D
PANC1+
PSC-control +PBS
PSC-KRASG12D +PBS
PSC-control +GEM
PSC-KRASG12D +GEM
Tumor Weight (g)
ns
**
PANC1+
PSC-control+ GEM
PSC-KRASG12D+GEM
PSC-control+ PBS
PSC-KRASG12D+PBS
E
PSC NC +GEM
PSC-KRASG12D +GEM
PSC-control +PBS
PSC-KRASG12D +PBS
Picrosirius Red
CD31
αSMA
% of Positive Areas
per 100× field
PANC1+
control+ GEM
KRASG12D+GEM
control+ PBS
KRASG12D+PBS

Figure S2. PSCs overexpressing KRAS^{G12D} promote collagen deposition and increases resistance to chemotherapy.

(A) Representative images and statistical analysis of tumor in NCG mice subcutaneously bearing BxPC3 and PSC-KRAS^{G12D} or control cells (n = 5, ns: not significant, two-tailed t-test). (B) Representative picrosirius red staining images and statistical analysis of percentage of positive areas of tumor in two groups in A (n = 5, three fields of view were selected for each tumor tissue, ****P < 0.0001, two-tailed t-test) (scale bars: 100×: 50 μm). (C) KEGG pathway from RNA sequencing between PSC-KRAS^{G12D} and control cells. (D) Representative images and statistical analysis of tumor in NCG mice subcutaneously bearing PANC1 and PSC-KRAS^{G12D} or control cells administered with gemcitabine (20 mg/kg) or PBS every three days (group treated with PBS n = 6, group treated with gemcitabine n = 5). (E) Representative images and statistical results of picrosirius red staining, CD31 and αSMA of tumor in each group (group treated with PBS n = 6, group treated with gemcitabine n = 5, three fields of view were selected for each tumor tissue, *P < 0.05, **P < 0.01, ****P < 0.0001, two-tailed t-test) (scale bars: 100×: 50 μm).

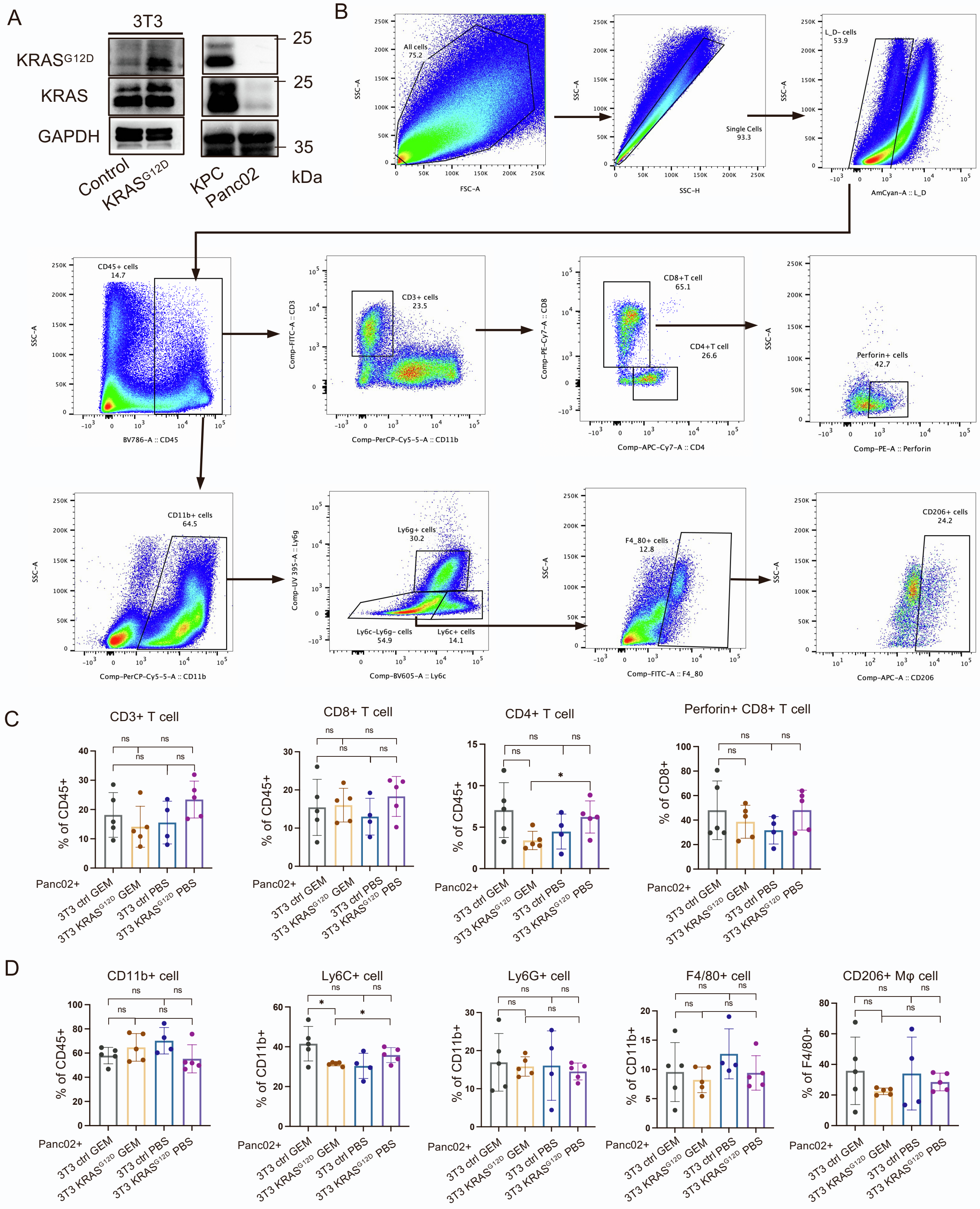

Figure S3. The overexpression of the KRAS^{G12D} protein in 3T3 cells has a minimal impact on the immune cell components in tumor.

(A) Western blot analysis of KRAS^{G12D} and KRAS expression in 3T3-KRAS^{G12D} and control cells. (B) Representative flow cytometry dot plots of T and myeloid cells. (C) Statistics of percentage of T cells (n = 4 for Panc02 and 3T3 PBS treated group and n = 5 for other groups, **P* < 0.05, ns: not significant, two-tailed t-test). (D) Statistics of percentage of myeloid cells (n = 4 for Panc02 and 3T3 PBS treated group and n = 5 for other groups, ns: not significant, two-tailed t-test).

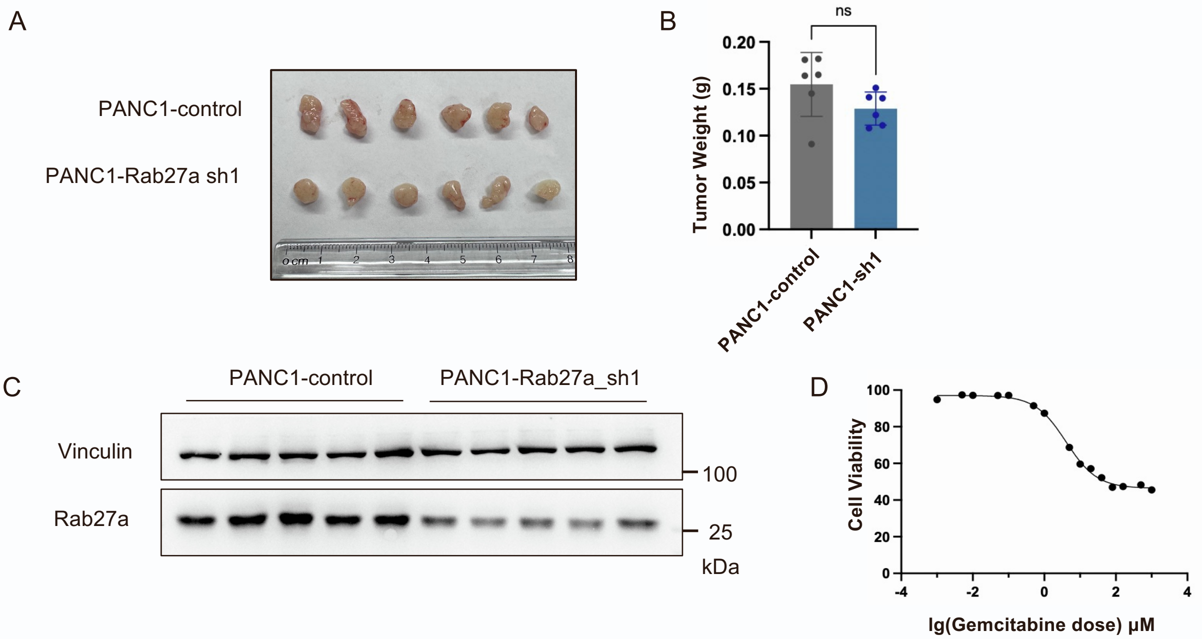

Figure S4. Blocking the secretion of EVs has no influence on tumor growth.

(A, B) Representative images and statistical results of tumor subcutaneously injected PANC1 control or PANC1-Rab27a_sh1 cell into the flank of NCG mice. (n = 6, ns: not significant, two-tailed t-test). (C) Western blot analysis Rab27a expression of tumor beared with PANC1 control or PANC1-Rab27a_sh1 cell (n = 5). (D) IC_{50} analysis of PANC1 cell treated with gemcitabine for 48 h.

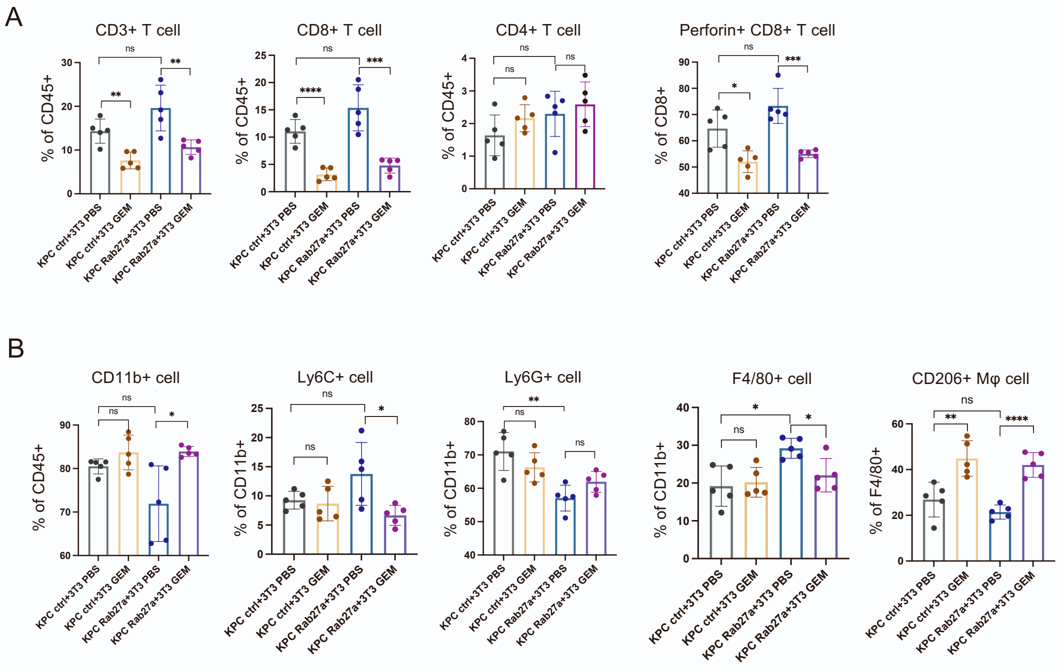

Figure S5. Reducing EVs release slightly affects specific myeloid cells within the tumor immune component.

(A) Statistics of percentage of T cells (n = 5, ***P* < 0.01, ****P* < 0.001, *****P* < 0.0001, ns: not significant, two-tailed t-test). (B) Statistics of percentage of myeloid cells (n = 5, **P* < 0.05, ***P* < 0.01, ****P* < 0.001, *****P* < 0.0001, ns: not significant, two-tailed t-test).

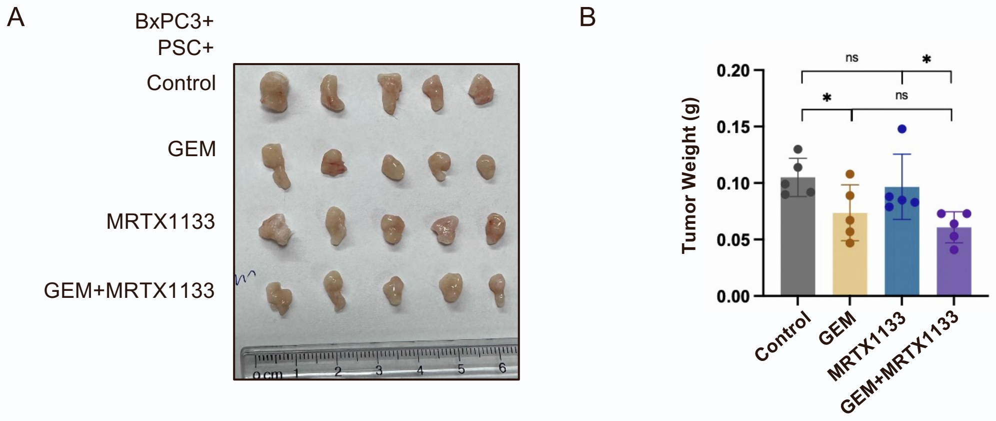

Figure S6. KRAS^{G12D} inhibitor slightly influences KRAS^{WT} tumor growth.

(A, B) Representative images and statistical results of tumor subcutaneously injected BxPC3 and PSC cells into the flank of NCG mice administered with gemcitabine (20 mg/kg), MRTX1133 (10 mg/kg) or their combination every three days (n = 5, *P < 0.05, ns: not significant, two-tailed t-test).

A

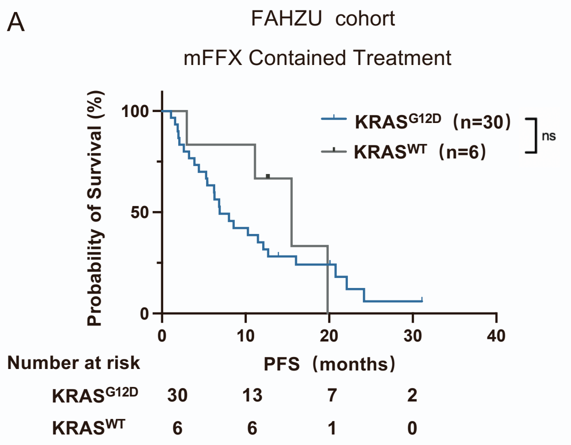

Figure S7. Patients with KRAS^{G12D} mutation have no survival influence on mFFX-containing treatment in PDAC.

(A) Survival curve of progression free survival of patients with KRAS^{G12D} mutation (n = 30) and KRAS^{WT} mutation (n = 6) who were treated with mFFX-contained regime (ns: not significant, Kaplan–Meier method and a Gehan-Breslow-Wilcoxon test).

Table S1. Basic characteristics of patients with KRAS^{G12D} or KRAS^{WT} mutation

	KRAS^{G12D} n=59	KRAS^{WT} n=15	P value
**Gender**			0.506
Male, n (%)	36(61)	9(60)	
Female, n (%)	23(39)	6(40)	
**Age, years**			0.943
>60, n (%)	37(63)	8(53)	
≤60, n (%)	22(37)	7(47)	
**BMI, kg/m2**			0.439
<18.5, n (%)	4(7)	1(7)	
18.5–23.9, n (%)	34(58)	6(40)	
>23.9, n (%)	21(36)	8(63)	
**Tumor location**			0.868
Proximal (Head, Neck or Uncinated process)	34(58)	9(60)	
Distal (Body or Tail)	25(42)	6(40)	
**Tumor size**			0.442
≥4cm, n (%)	22(37)	4(27)	
<4cm, n (%)	37(63)	11(73)	
**Lymph nodes metastasis**			0.868
Yes, n (%)	25(42)	5(33)	
No, n (%)	34(58)	10(67)	
**TNM stage**			0.235
I, n (%)	16(27)	6(40)	
II, n (%)	9(15)	3(20)	
III, n (%)	0(0)	0(0)	
IV, n (%)	34(58)	6(40)	
**Serum CA19-9, U/mL**			0.111
≥37, n (%)	46(78)	8(53)	
<37, n (%)	13(22)	7(47)	
**Serum CA12-5, U/mL**			0.656
≥60, n (%)	9(15)	1(7)	
<60, n (%)	50(85)	14(93)	
**Serum CEA, U/mL**			0.919
≥5, n (%)	19(32)	4(27)	
<5, n (%)	40(68)	11(73)	
**Recurrence**			0.217
Yes, n (%)	48(81)	10(67)	
No, n (%)	11(19)	5(33)	
